# Molecular Targeting of the Most Functionally Complex Gene in Precision Oncology: p53

**DOI:** 10.3390/cancers14215176

**Published:** 2022-10-22

**Authors:** Douglas W. Brown, Perrin H. Beatty, John D. Lewis

**Affiliations:** 1 Department of Oncology, University of Alberta, Edmonton, AB T6G 2E1, Canada; 2 Entos Pharmaceuticals, Unit 4550, 10230 Jasper Avenue, Edmonton, AB T5J 4P6, Canada

**Keywords:** genetic therapy, p53, p53 and signaling, solid tumors, cancer treatment

## Abstract

**Simple Summary:**

Precision medicine is a powerful treatment modality for controlling tumor growth. However, given the substantial variability between cancer patients, identifying suitable targets is no easy task. Tumor protein p53 represents one of the most promising anti-cancer drug targets due to its high mutation rate and critical role in tumorigenesis. Despite this, current treatment strategies targeting p53 signaling have only seen modest clinical success presumably due to the complex signaling network surrounding p53 enabling cancer cells to have the ability to adapt to each treatment strategy. This review will focus on the multiple approaches to target p53 and will touch on some of the limitations that may be hindering their clinical success.

**Abstract:**

While chemotherapy is a key treatment strategy for many solid tumors, it is rarely curative, and most tumor cells eventually become resistant. Because of this, there is an unmet need to develop systemic treatments that capitalize on the unique mutational landscape of each patient’s tumor. The most frequently mutated protein in cancer, p53, has a role in nearly all cancer subtypes and tumorigenesis stages and therefore is one of the most promising molecular targets for cancer treatment. Unfortunately, drugs targeting p53 have seen little clinical success despite promising preclinical data. Most of these drug compounds target specific aspects of p53 inactivation, such as through inhibiting negative regulation by the mouse double minute (MDM) family of proteins. These treatment strategies fail to address cancer cells’ adaptation mechanisms and ignore the impact that p53 loss has on the entire p53 network. However, recent gene therapy successes show that targeting the p53 network and cellular dysfunction caused by p53 inactivation is now possible and may soon translate into successful clinical responses. In this review, we discuss p53 signaling complexities in cancer that have hindered the development and use of p53-targeted drugs. We also describe several current therapeutics reporting promising preclinical and clinical results.

## 1. Introduction

Despite decades of research and rapidly advancing treatment modalities, cancer remains the second leading cause of premature death worldwide [1,2]. Cells are constantly exposed to exogenous stressors that can cause DNA mutations, such as ionizing radiation (UV, X-rays, Gamma) and carcinogenic chemicals. Additionally, replication errors and defective DNA repair mechanisms can gradually accumulate mutations and cause cancer over time [3,4]. Multicellular organisms have evolved redundant tumor-suppressive functions to prevent malignant transformation, such as cellular senescence and apoptosis. However, cancerous cells can avoid, overcome, or override these suppressive mechanisms [5,6]. The Hanahan and Weinberg hallmarks of cancer illustrated some of these abilities, such as resistance to apoptotic stimuli, limitless proliferation, immune avoidance, persistent mutation accumulation, metastasis, and many more [7,8,9,10,11,12]. These hallmarks are likely driven by natural selection, as cells developing these characteristics show a survival and growth advantage facilitating their propagation [13,14]. Cancer cells that adapt to avoid death by conventional genotoxic treatments such as chemotherapy and radiation can yield treatment-resistant progeny [15,16,17,18]. Therefore, we can classify cancer as a disease of dysfunction and chaotic cellular signaling, and this raises an important question; how do we treat chaos? One strategy has been developing precision treatments based on the unique gene signatures of individual cancers. Treatments targeting specific molecular targets have been used for many years with varying response rates depending on the target and cancer subtype [10,19,20,21,22,23]. Most preclinical drug discovery programs aim to find a single target with high anti-tumor efficacy in multiple cancer subtypes. However, as researchers uncover more cancer cell signaling complexities, this objective seems unlikely to be effective. Despite this, precision medicine is a growing field with the discovery of novel targets and treatments at a rapid rate [21,24,25,26,27]. This review article discusses the targeted therapy approaches of one of the most promising yet complex anti-cancer drug targets, tumor protein p53.

## 2. Tumor Protein p53

The tumor suppressor protein p53 has been called the ‘guardian of the genome’ [28] due to its signaling function and other newly-discovered properties in multiple anti-cancer pathways [28,29,30,31,32,33,34]. We briefly touch on the role of p53 in tumorigenesis regarding potential treatment strategies as p53 signaling has been reviewed extensively elsewhere [35,36,37]. p53 predominantly functions as a master regulator of cellular stress, where stressors such as DNA damage, oxidative stress, oncogene activation, and cell cycle progression promote p53 activation and accumulation. Activated-p53 functions as a transcription factor that upregulates target genes’ expression to facilitate cell cycle arrest, DNA repair, senescence, and apoptosis [29]. In unstimulated cells, the cellular level of functional p53 protein is usually kept low through its interaction with the E3-ubiquitin ligase, mouse double minute homolog 2 (MDM2). The physical interaction between p53 and MDM2 prevents p53 from activating transcription, while MDM2-mediated ubiquitination of p53 tags it for proteasomal degradation [38,39]. A structurally homologous protein, MDMX/MDM4, has a similar role to MDM2 in negatively regulating p53 activity, except MDMX is not an E3-ubiquitin ligase and instead inhibits p53 transcriptional activity by physical association [40,41]. However, there is evidence that supports a model in which MDMX enhances the ability of MDM2 to ubiquitinate p53 [42].

**Figure 1:**
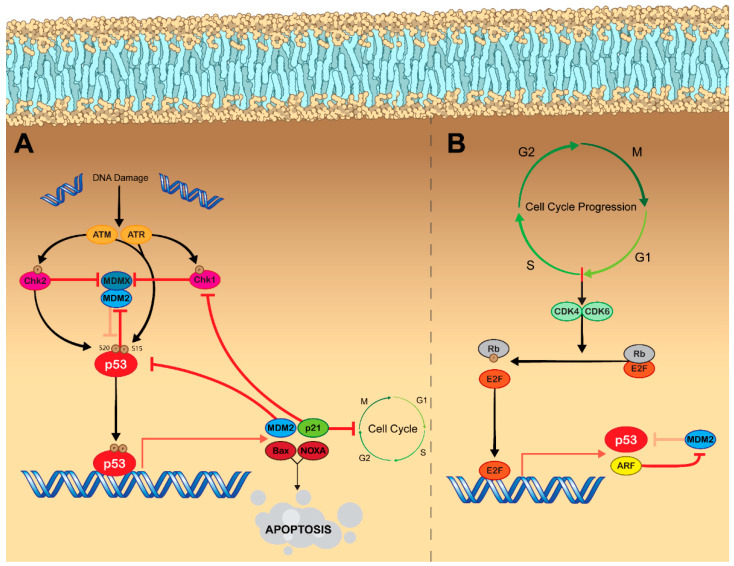
Mechanisms of p53 activation. (**A**) DNA damage results in p53 activation via engaging the DDR pathway. Following detection of single stranded or double stranded DNA breaks, the kinases ATM and ATR become activated. Both ATM and ATR phosphorylate p53 at Ser15. ATM also phosphorylates Chk2, which phosphorylates p53 at Ser20, while ATR phosphorylates Chk1. Phosphorylation of p53 at Ser15 and Ser20 causes it to be released from its negative regulator, MDM2. Additionally, Chk2 and Chk1 both inhibit MDMX, which functions to enhance the E3 ligase activity of MDM2 and inhibit p53. With p53 now released from MDM2, it functions as a transcription factor resulting in the expression of various tumor suppressive genes. For example, Bax and NOXA are proapoptotic proteins, while p21 functions to inhibit cell cycle progression. p53 also plays a role in regulating its own activity by facilitating the expression of more MDM2, while p21 acts to inhibit further DDR signaling by blocking the activity of Chk1. (**B**) Cell cycle progression also leads to increased activity of p53. As cells prepare to enter the S phase of the cell cycle, the kinases CDK4 and CDK6 become activated. CDK4/CDK6 phosphorylate the cell cycle inhibitor protein Rb, which causes it to dissociate from E2F. E2F is now free from inhibition and functions as a transcription factor resulting in the expression of genes that facilitate cell cycle progression. E2F also facilitates the expression of p53 as well as ARF, which functions as an MDM2 inhibitor—therefore engaging p53 signaling. E2F can also facilitate nuclear accumulation of p53 by blocking its nuclear export signal. Legend: Arrows represent activation pathways, hammers represent inhibition, faded hammers represent inhibitory pathways that are being repressed by the signal pathway. Abbreviations: ATM, ataxia–telangiectasia mutated kinase; ATR, ataxia-telangiectasia, and Rad3-related kinase; Chk1/Chk2, checkpoint kinases 1&2; MDM2, mouse double minute 2 homolog; MDMX mouse double minute X; CDK4/CDK6, cyclin-dependent kinases 4&6; Rb, retinoblastoma protein; E2F, E2 factor; ARF, alternative reading frame (p14).

Genotoxic stress, such as chemotherapy and ionizing radiation, can also activate p53, leading to its accumulation and subsequent transcription of p53-regulated genes due to the convergence of the DNA damage response (DDR) pathway and p53 signaling [43,44,45,46,47]. DNA damage activates ataxia–telangiectasia mutated (ATM) kinase and ataxia–telangiectasia and Rad3-related (ATR) kinase. These kinases, in turn, phosphorylate the checkpoint kinases Chk2 and Chk1, respectively [48]. ATM and ATR both phosphorylate p53 at Ser15, while Chk2 phosphorylates p53 at Ser20. ATM also phosphorylates MDM2 at Ser395 [49] In turn, these phosphorylation events cause p53 to be released from MDM2 [30,50,51,52]. DNA damage also directly affects MDMX, as ionizing radiation results in phosphorylation by Chk2, leading to MDM2-dependent degradation [53,54]. Additionally, phosphorylation by Chk1 in response to ultraviolet radiation results in MDMX nuclear export by enhancing its binding to cytoplasmic 14-3-3γ [55]. These actions cause p53 to accumulate, which enables the transcription of tumor suppressive p53-target genes. p53 is then able to negatively regulate its own activity by inducing MDM2 expression (Figure 1A) [56]. Negative regulation is also achieved via the expression of wild-type p53-induced phosphatase 1 (Wip1), which dephosphorylates MDM2 at Ser395 and increases its affinity for p53 to aid in reducing the cell to baseline p53 levels [57].

Cell cycle progression indirectly leads to activation of p53, as transition through the G1 phase to S phase results in the activation of the cyclin-dependent kinases, CDK4 and CDK6, which phosphorylate retinoblastoma protein (pRb) causing dissociation of the pRb–E2F complex. E2F, now free from inhibition, activates the transcription of multiple genes involved in the cell cycle progression from G1 phase to S phase. Interestingly, E2F also facilitates the transcription of ARF (p14^ARF^ in humans and p19^ARF^ in mice) [58], which inhibits the ubiquitin ligase activity of MDM2, thereby resulting in p53 accumulation [59]. Additionally, E2F can directly bind to p53 and block a nuclear export signal causing it to accumulate in the nucleus (Figure 1B) [60]. p53 functioning as a transcription factor facilitates the expression of p21, which inhibits the activity of CDK2, CDK4, and CDK6, thus causing G1, G2, or S phase arrest and therefore halts the cell cycle [61].

Activated p53 oligomerizes to form tetramers, which stabilize the binding interaction with p53-response elements in DNA and enable the expression of p53-regulated genes to initiate various cellular programs [62]. p53 regulates thousands of genes involved in cell cycle arrest, senescence, apoptosis, autophagy, metabolism, and DNA repair [63]. Cell fate decisions depend on the degree of p53-inducing stress. More importantly, the decision whether to facilitate growth arrest or apoptosis in response to DNA damage appears to depend on overall p53 levels, as p53 simultaneously expresses growth arrest proteins p21 and polo-like kinase 3 (PLK3) as well as proapoptotic proteins PIG3 and APAF1. Cell cycle arrest is initiated immediately following stimulation with low p53 levels, however, prolonged high levels of p53 are required for PIG3 and APAF1 to reach the levels required to overcome the apoptotic threshold and commit cell to cell death [64,65]. In the early stages of growth arrest, p53 also facilitates the expression of DNA repair enzymes, these include the nucleotide excision repair factors p48 and XPC, as well as the base excision repair enzymes 8-oxoguanine glycosylase and MUTYH [66]. Growth arrest works cooperatively with DNA repair to first prevent the damage from propagating but then give the cell an opportunity to mend the damage. If these two functions are unsuccessful in halting the DDR, p53 levels will continue to rise and therefore cause increased expression of proapoptotic proteins until they reach critical levels required for cell death [64]. Persistent DNA damage can also result in p53-dependent activation of cellular senescence to cause irreversible cell cycle arrest without apoptosis induction. As mentioned previously, p53 facilitates the expression of p21, which functions as a CDK inhibitor, therefore halting cell cycle progression [67]. Interestingly, p21 may be partially responsible for regulating the decision to undergo senescence or apoptosis as p21 has been found to negatively regulate caspase and Jun N-terminal kinase (JNK) activity in response to DNA damage, thus preventing apoptosis induction [68]. Autophagy represents another cell fate decision regulated by p53 in response to DNA damage [69]. In the context of p53 signaling, autophagy primarily functions as a tumor suppressor by facilitating the degradation of damaged cellular components to regulate nutrient cycling under stressful metabolic conditions [70]. p53 facilitates the expression of tuberous sclerosis complex 2 (TSC2) and AMP-activated protein kinase (AMPK), which inhibit the activity of mammalian target of rapamycin (mTOR), a key negative regulator of autophagy [71]. A direct link between p53 and autophagy exists, whereby p53 induces the expression of the autophagy promoting genes, damage-regulated autophagy modulator (DRAM) [72], the autophagy-related genes, Atg4a, Atg4c, and Atg7, as well as Ulk1 and Ulk2 [73]. Autophagy exhibits indirect negative feedback on p53 signaling, presumably by reducing oxidative stress that may be inducing p53 activity [74].

Since its critical role as a tumor suppressor was uncovered [75,76,77,78,79], there has been a significant research effort being directed to understanding the complexities of p53 signaling in cancer and in normal cell biology [33]. Most of this research has been focused on understanding p53 signaling at the protein level and not on the transcriptional regulation of p53 itself [34]. However, a few transcription factors have been found to directly bind to and regulate the p53 promoter. Positive regulators such as Myc [80] and HoxA5 [81] are interesting as oncogenic Myc signaling is intricately linked to cancer progression [82] and HoxA5 appears to be an important tumor suppressor [83,84]. Understanding the regulation of p53 transcription in the context of cancer is not a straightforward topic due to the complex interplay between transcription factors, epigenetic mechanisms, and miRNAs, as well as the impact mutant p53 (mup53) has on its own regulation [34].

## 3. p53 and Cancer

p53 is the most mutated protein in cancer, with nearly 50% of all tumor cells displaying some form of p53 mutation [31], with mutation being used to characterize cancer progression [85,86,87]. Even in the absence of a detectable mutation, some estimates predict that 80% of tumors have impaired p53 function, caused by epigenetic silencing or overexpression of negative regulators such as MDM2 [88,89]. Most p53 mutations are missense mutations that typically lead to the accumulation of the mutant protein [90]. Interestingly, these missense mutations tend to cluster into ‘hotspots,’ with ~25% of all mutations occurring at 1 of 6 codons within the p53 DNA-binding domain [31,88]. Missense mutations are classified as either DNA contact mutants (R248, R273) or p53 conformation mutants (R175, G245, R249, R282) [31,91]. The type of p53 mutation can have vastly different consequences on the cell, with these hotspot mutations being associated with mup53 oncogenic gain of function (GOF) tumor-promoting capabilities. If only one p53 allele is mutated, a dominant negative (DN) effect may occur, where the mup53 can bind and sequester wild-type (WT) p53 and block normal function [90]. Additionally, the conformation mutation mup53^R175H^ has been found to bind and sequester the tumor suppressors p73 and p63 [92], both of which facilitate the expression of genes similar to p53 [93]. GOF mup53 plays a pivotal role in tumorigenesis and cancer progression. Mice possessing germline GOF mutations in one p53 allele develop unique tumors with high metastatic capacity when compared to p53^+/−^ and p53^−/−^ mice [94,95,96]. GOF mup53 has been demonstrated to affect multiple oncogenic signaling pathways, upregulating various stem cell markers in colorectal cancer cells [97] and increasing human epidermal growth factor receptor 2 (HER2) expression in breast cancer cells [98].

p53 is implicated in virtually all the Hanahan and Weinberg “hallmarks of cancer.” Activation of p53 in response to DNA damage, proliferation, and oxidative stress leads to cell cycle arrest and activation of apoptotic pathways [29]. p53 is intimately linked to cellular metabolism and functions to regulate nutrient availability and glycolysis [99,100]. Additionally, WT p53 functions to prevent metastasis in part due to the expression of miRNAs that regulate the epithelial to mesenchymal transition [101,102]. This could possibly explain why most advanced cancers present with a mutation in p53 [86,87]. p53 is activated in response to proliferative signals brought on by inflammation, and proinflammatory cytokines possess the ability to directly regulate p53 expression and accumulation [103,104]. The master regulator of inflammatory signals, NF-κB, is activated by various cytokines and facilitates the transcription of anti-apoptotic proteins, cytokines, and growth factors; all of which lead to enhanced tumorigenesis [105,106]. Despite the opposing functions of NF-κB and p53 on tumor progression, NF-κB has been found to directly bind to the p53 promoter and facilitate its expression, establishing a potential mechanism for negative regulation of tumorigenic inflammatory signals [107]. Clearly, the importance of p53 dysfunction in tumorigenesis cannot be overlooked. As such, a handful of novel treatments are currently under development with the intention of targeting p53 and p53 signaling.

## 4. Restoring Endogenous p53 Signaling

**Figure 2:**
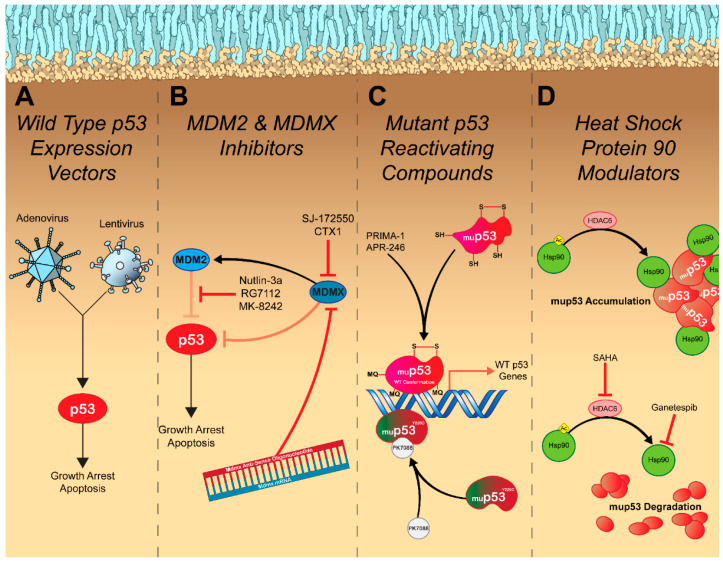
Therapeutic strategies for targeting p53. (**A**) Viral expression vectors (adenovirus and/or lentivirus) that encode the WT p53 protein, hijack host (cancer) cell machinery and force them to produce functional p53 protein. This leads to activation of canonical p53 signaling, resulting in growth arrest and apoptosis induction. (**B**) MDM2 and MDMX inhibitors function to block the negative regulation of p53 by these proteins, resulting in p53 release and activation of tumor suppressive pathways such as growth arrest and apoptosis. In addition to small molecule inhibitors of the p53 binding site on MDM2 and MDMX, antisense oligonucleotides can be directed against Mdmx mRNA by targeting the boundary of exon 6 resulting in downregulation of MDMX. (**C**) Mutations of p53 protein may cause conformational changes in the DNA binding domain. Compounds that stabilize the mutant protein so that the WT conformation of the DNA binding domain can be restored are able to facilitate the expression of WT p53 target genes resulting in growth arrest and apoptosis. These include PRIMA-1/APR-246, which following conversion into methylene quinuclidinone (MQ) can covalently attach to cysteine residues, which stabilizes the binding of mup53 to the WT proteins DNA response elements. Additionally, mutations can create novel binding domains on the mup53 protein. The Y220C mutation generates a binding pocket on mup53 that when bound by the small molecule PK7088 stabilizes the mutant protein in its WT conformation enabling binding to DNA response elements. These compounds enable restoration of WT p53 transcriptional functions resulting in cell cycle arrest and apoptosis. (**D**) Heat shock protein 90 (Hsp90) functions to stabilize mup53, preventing its degradation. Addition of the Hsp90 inhibitor, ganetespib, blocks this interaction and facilitates degradation of mup53. This can also be achieved by inhibiting histone deacetylase activity with suberoylanilide hydroxamic acid (SAHA), which causes Hsp90 to remain acetylated, which blocks its chaperone activity. Legend: Arrows represent activation pathways, hammers represent inhibition, and faded hammers represent inhibitory pathways that are being repressed by the signal pathway.

Tumor suppressors evolved to prevent malignant transformation, with oncogenic stimuli leading to their activation. As such, cancer cells are constantly activating the pathways that evolved to trigger p53 signaling. However, with p53 function lost, they exist in a state of perpetual p53 activation. This led to the hypothesis that if normal p53 function were to be restored, cancer cells would be primed to arrest their growth and undergo apoptosis. Support for this method of treatment was strengthened following reports that replacement of WT p53 into p53-null cell lines resulted in arrested growth, increased apoptosis induction, and caused tumor regression in vivo [108,109]. p53 gene therapy was born in 1994 after Fujiwara, T. et al. [110] demonstrated that intratracheal administration of a retroviral vector expressing WT p53 suppressed the growth of established lung tumors in vivo. A small clinical trial was conducted in nine non-small cell lung cancer (NSCLC) patients and found that bronchoscopic administration of the retroviral-WT p53-vector suppressed tumor growth and increased the presence of the TUNEL apoptosis marker at follow-up [111]. A major limitation of this study was the use of the retroviral vector, which has significant safety concerns, mainly, the risk of insertional mutagenesis [112]. To overcome the safety concerns, studies examining the use of a safer adenoviral WT p53 expression vector (Ad-p53) were conducted in multiple cancer subtypes and demonstrated similar effects—apoptosis induction and arrested growth in vitro and in vivo (Figure 2A), with genotoxic combination therapy resulting in a synergistic effect [113,114,115]. Clinical trials with this treatment have produced mixed results. Intratumoral injection of Ad-p53 in NSCLC patients resulted in a clinical response in 72% of patients [116], and intratumoral injection into patients with head and neck cancer resulted in a clinical response in 47% of patients (Table 1) [117]. These response rates could be strengthened by combining treatment with radiation therapy [118,119]. Because of the encouraging response in patients with head and neck cancer, in 2003, China granted regulatory approval for the Ad-p53 treatment, making it the first gene therapy product ever approved [120]. Thousands of cancer patients have received some form of restorative p53 gene therapy with varying success rates. It appears that restoring endogenous p53 activity via genetic means represents a safe means for controlling tumor growth, however, some form of genotoxic combination therapy seems to be necessary to produce meaningful response rates. Unfortunately, the addition of Ad-p53 often does not produce a survival benefit when compared to the genotoxic treatment alone, which raises concerns about its potential use in the future [121]. The variation in response to treatment could potentially be due to the presence of different p53 mutations. Wang et al. [122] found that restoring endogenous p53 function in mice harboring the p53^R172H^ mutation (analogous to the R175H hot-spot mutation in humans) did not lead to tumor regression as it does in p53-null mice. Mutant p53^R172H^ was demonstrated to bind to the restored WT p53 and exert a dominant negative effect that prevented the expression of pro-apoptotic proteins but not proteins involved in cell cycle arrest [123]. Cancer cells with WT p53 would also be largely unaffected by p53 restoration, as the p53 signaling network is still relatively intact [124,125]. Unfortunately, it appears that the intricacies surrounding p53 inactivation are too complex to be overcome by simply replacing WT p53. However, with the recent success of immunotherapies in controlling tumor growth, there has been slightly renewed interest in Ad-p53 treatments as it pertains to combination therapy. Adenoviruses are highly immunogenic [126,127], and though this is regarded as a negative aspect of treatment, as humoral immune responses to the viral vector may hinder repeat dosing, immune stimulation may be advantageous for cancer therapy. Dendritic cells have been demonstrated to uptake apoptotic bodies and illicit potent anti-tumor responses [128,129]. A potential synergy exists between the immune stimulation of the adenoviral vector and the generation of immunogenic apoptotic bodies caused by the WT p53 gene. Ad-p53 treatment has been demonstrated to influence various immune signaling pathways, resulting in an increased CD8 T cell response that correlates with enhanced immune checkpoint inhibitor efficacy [130,131]. Clinical trials are being conducted to evaluate the feasibility of combining Ad-p53 therapy with anti-PD-1 or anti-PD-L1 checkpoint inhibitors [NCT03544723].

Despite the limitations surrounding restorative p53 gene therapy, it does indicate that there is potential that p53 reactivation could result in more meaningful clinical responses if a targeted approach is taken. In the case of cancer cells with WT p53 that present with overexpression of a negative regulator, there are several treatment modalities being explored for each specific indication. Perhaps the most promising and extensively studied are inhibitors of the MDM2-p53 interaction. The discovery that the small molecule nutlin-3a could bind to MDM2 and release p53 from its inhibition resulting in apoptosis and hindered tumor growth in vivo [132] spurred the development of multiple small molecule drugs that can also inhibit this interaction [133,134,135,136,137]. In patients with liposarcoma, the MDM2 antagonist, RG7112, was able to facilitate p53 accumulation and control tumor growth (Figure 2B and Table 1). However, 40% of patients presented with serious adverse events, primarily neutropenia and thrombocytopenia [138], presumably due to increased apoptosis in hematopoietic progenitors in which the MDM2-p53 axis plays an essential role in their differentiation [139]. In addition to safety concerns, MDM2 inhibitors are also subject to resistance mechanisms such as overexpression of efflux pumps and mutation to p53, which further hinders their clinical success [140]. Of the MDM2 antagonists currently in clinical trials, most compounds display similar efficacy with newer generation drugs displaying slightly improved safety profiles [138]. Due to the early clinical stages of these compounds, few have evaluated clinical responses to treatment and overall survival. One compound, MK-8242, tested in patients with liposarcoma resulted in a median progression-free survival of 237 days (Table 1). Unfortunately, due to the small cohort size, a proper control was not included in this study and the authors compared their median progression-free survival to that of another study that resulted in a median progression-free survival of 126 days in patients with liposarcoma treated with a CDK4 inhibitor [141]. Evidently, larger clinical studies with better controls are necessary to determine the validity of this treatment approach.

The variability in therapeutic responses to MDM2 antagonists can be partially explained due to the engagement of multiple p53-dependent pathways apart from apoptosis. Efeyan, A., et al. [142] demonstrated that nutlin-3a induced cell cycle arrest and senescence in fibrosarcoma cells. The authors also showed that senescence was induced in primary fibroblasts as well. This is important as senescent cells possess novel tumor-promoting capabilities due to the acquisition of the senescence-associated secretory phenotype (SASP)—a collection of soluble and insoluble markers that influence the tumor microenvironment with factors that are proinflammatory (cytokines/chemokines), matrix remodeling (MMP), or promote cell growth (growth factors) [143]. Nutlin-3a has also been demonstrated to facilitate p53-dependent autophagy by stimulating the expression of AMPK and DRAM [144]. Though autophagy possesses tumor suppressive functions in healthy cells, in cancer cells autophagy is tumorigenic by enabling cells to endure stressful conditions such as nutrient deprivation and oxidative damage [70]. It is possible that MDM2 antagonists produce poor clinical responses due to these indirect mechanisms of action. Interestingly, combination treatment with nutlin-3a and a Wip1 inhibitor significantly increases apoptosis induction when compared to monotherapy with either compound [145]. As nutlin-3a is a reversible MDM2 antagonist [146], Wip1 inhibition may prevent negative regulation by MDM2 at lower drug concentrations and enable prolonged p53 signaling, which is required to express sufficient amounts of proapoptotic proteins to overcome the apoptotic threshold [64,65].

Targeting the MDMX-p53 interaction represents an alternative strategy for restoring p53 activity as it may result in fewer side effects than MDM2 antagonists. Hematopoietic stem cells recover from short-term p53 restoration in mice lacking MDMX, presumably due to functional MDM2 retaining the ability to regulate p53 activity [147]. MDMX targeted treatments are still in preclinical stages of development, though a handful of promising compounds have been identified [148]. Small molecule antagonists targeting the p53 binding site of MDMX, such as SJ-172550 and CTX1, typically lead to accumulation of the p53 protein and expression of p53-regulated genes resulting in similar effects to MDM2 antagonists: growth arrest and apoptosis (Figure 2B) [148]. Though reducing toxicity associated with MDM2 inhibition was the primary driving force behind the development of MDMX antagonists, these compounds display potent synergism when combined [149,150]. One of the most exciting MDMX inhibitors is an antisense oligonucleotide (ASO) directed against full-length MDMX. Dewaele et al. [151] discovered that adult tissue lacked full-length MDMX primarily due to inefficient splicing of Mdmx mRNA at exon 6, which does not occur in cancer cells. Therefore, they designed a cancer-selective MDMX-ASO that targets the exon–intron boundary of exon 6 and leads to a reduction in MDMX protein levels, impaired in vitro growth, and increased apoptosis induction as well as attenuated the growth of both melanoma and large B cell lymphoma patient-derived xenografts (Figure 2B). Clinical data will be necessary to indicate if MDMX inhibitors provide any advantage over targeting MDM2. Unfortunately, it is likely that MDMX inhibitors will be subject to similar resistance mechanisms as MDM2 inhibitors and may have unique safety concerns yet to be uncovered.

Cancer cells possessing mutant forms of p53 can be targeted using compounds that reverse the conformational changes acquired by mutation, thus restoring p53 function. The most clinically advanced compound, PRIMA-1, can preferentially inhibit the growth of cancer cell lines with the R273H DNA contact mutant or the R175H conformation mutant [152]. Upon entry into cancer cells, PRIMA-1 is converted to methylene quinuclidinone (MQ) that covalently binds to the p53 core via alkylation of cysteine residues. This may prevent the formation of disulfide bonds that fix mup53 in an unfolded confirmation [153]. Additionally, MQ alkylation was recently demonstrated to stabilize mup53 binding to DNA, therefore enabling expression of WT p53 genes (Figure 2C) [154]. A recent phase II clinical trial examined the efficacy of the PRIMA-1 derivative, APR-246 (eprenetapopt), in combination with the first-line therapy, azacytidine, in patients with acute myeloid leukemia or myelodysplastic syndromes with at least one p53 mutation (Table 1). APR-246 was well tolerated and displayed favorable response rates with a potentially increased survival when compared to previously published data on monotherapy with azacytidine alone [155]. Unfortunately, a phase III study with APR-246 in this patient population failed to display significant improvements in complete remission rate when compared to monotherapy with azacytidine (33.3% vs. 22.4%, respectively; *p* = 0.13) [156]. Neither of these studies were specific in their p53 mutation inclusion criteria, and therefore it is possible that targeting specific p53 mutants (R273 and R175) may improve treatment responses. Unfortunately, as the inclusion criteria for clinical studies becomes more specific for each p53 mutation, our ability to perform well-powered trials is reduced, which will further delay the entry of these compounds into the clinic.

Other mup53-selective compounds are in preclinical development. PK7088 targets cancer cells harboring a Y220C p53 mutation, the ninth most common p53 mutation. PK7088 binds mup53 in a pocket created by the Y220C mutation and slows its denaturation [157]. This results in a restoration of the WT p53 conformation, nuclear accumulation of p53, and the expression of p53-dependent genes, which facilitate cell cycle arrest and apoptosis induction (Figure 2C) [158]. Other indirect methods of restoring the WT p53 conformation have also been discovered. The small molecule chetomin was identified in a high-throughput screen and preferentially facilitated p53 gene expression and apoptosis induction in cell lines and tumors containing the R175H mutation. Interestingly, chetomin does not bind mup53 and instead increases the binding affinity of Hsp40 for mup53^R175H^. Hsp40 can then bind to mup53^R175H^ and stabilize the unfolded protein, restoring its function [159]. The promising preclinical results from these compounds are encouraging and indicate that we may soon have multiple p53 restorative therapeutics entering the clinic, provided they produce meaningful clinical responses [160].

## 5. Inhibiting Oncogenic Gain of Function p53 Signaling

As if understanding endogenous p53 signaling was not complicated enough, even understanding p53 mutation is not a straightforward topic. Contrary to most tumor suppressor genes that undergo a loss-of-function following mutation, p53 can lose its endogenous function while simultaneously gaining novel tumor-promoting capabilities. DNA contact mutants and conformational mutants alter the DNA binding domain, which can alter the DNA elements recognized by p53 and result in the expression of tumorigenic genes involved in apoptosis resistance, proliferation, angiogenesis, and metastasis [90]. Additionally, mup53 may associate with other oncogenic proteins and facilitate activation of their tumorigenic pathways [161].

In some cases, cancer cells possessing a GOF mutation in p53 are dependent on it for their survival, and knockdown of mup53 abrogates tumorigenic potential in vitro and in vivo [162,163]. In a proof-of-concept experiment, Alexandrova et al. [164] demonstrated that genetically ablating mup53^R248Q^ slowed tumor growth and prolonged survival. The authors also demonstrated that Hsp90 stabilized mup53, and adding the Hsp90 inhibitor, ganetespib, increased median survival in tumor-bearing mice possessing either the R248Q or the R172H (R175H in humans) p53 mutants by 59% and 48%, respectively, with no effect on p53-null mice. Ganetespib-mediated inhibition of Hsp90 results in selective degradation of mup53 that is accompanied by apoptosis induction, thus providing evidence that elimination of GOF mup53 represents a viable treatment option (Figure 2D). Histone deacetylase inhibitors (HDACi) represent an alternative strategy to indirectly enhance mup53 degradation. The HDACi, suberoylanilide hydroxamic acid (SAHA), was found to inhibit HDAC6 and as a result, Hsp90 remains acetylated at K294, which inhibits its chaperone activity. In turn, mup53 is released from Hsp90 and degraded resulting in apoptosis induction selectively in cancer cells harboring mup53 (IC50 values are >100-fold higher in cells with WT p53) (Figure 2D) [164,165]. Unfortunately, clinical trials with both ganetespib and SAHA have not been very promising (Table 1). Patients in these trials were not selected based on their p53 status and therefore it is entirely possible that the efficacy of these two compounds can be improved by altering the inclusion criteria [166,167].

Perhaps one of the more obvious strategies to inhibit GOF mup53 would be via knockdown with siRNA. However, there is potential that siRNA may still recognize WT p53 in healthy tissue leading to off-target effects. Martinez et al. [168] demonstrated that siRNAs can be designed to preferentially recognize single nucleotide changes in p53 mRNA by ensuring that the ninth nucleotide in the siRNA contained the mutated base pair. Recently, Ubby et al. [169] generated a panel of siRNAs targeting each GOF mup53 and used lipid nanoparticles to deliver them to subcutaneous tumors, where they abrogated tumor growth. Though clearly still in its infancy, the preclinical data have indicated that eliminating mup53 can have potent anti-tumor effects.

## 6. Exploiting Dysfunctional p53 Signaling

Rather than trying to correct the problems brought on by p53 mutation, an alternative approach is to capitalize on it and utilize the dysfunction for treatment options. The earliest example of this is ONYX-015, a replication-competent adenovirus that selectively infects and lyses cancer cells lacking functional p53. By removing the E1B gene that normally functions to inactivate p53 in infected cells [170], the adenovirus would only be able to replicate in cells lacking p53 as cells with a functional copy of p53 would block viral replication by arresting their growth or undergoing apoptosis [171]. However, subsequent reports found that viral replication was not restricted to cells lacking p53, instead, it showed a slight preference for replicating in cancer cells with mup53 [172,173,174,175]. Unfortunately, ONYX-015 failed clinical trials as a monotherapy, because although it had a favorable safety profile, it demonstrated minimal antitumor efficacy [176,177]. However, ONYX-015 was able to sensitize cancer cells to chemotherapy [177,178,179], which reinvigorated interest in the treatment and eventually led to regulatory approval of a similar oncolytic virus (H101) in China [180].

GOF mutations in p53 can result in unique gene expression signatures relative to WT p53 possessing counterparts [90]. Because of this, mup53 may facilitate the expression of druggable genes offering a novel approach for selectively targeting cancer. For example, mup53 can bind and sequester p73, which enables NF-Y to be released from p73 and facilitate the expression of platelet-derived growth factor receptor β (PDGFRβ). Consequentially, PDGFRβ then stimulates invasion and metastasis. However, this also enables the process to be blocked by adding PDGFRβ inhibitors such as imatinib and crenolanib [181]. Mutant p53 has also been demonstrated to directly bind to NF-Y and recruit p300 in response to DNA damage. This results in the expression of genes involved in cell cycle progression that would normally be inhibited in response to DNA damage [182]. P300 is a histone acetyltransferase, therefore making it a druggable target. In fact, a number of p300 (and CREB-binding protein) inhibitors are currently under development and clinical investigation for the treatment of multiple cancer subtypes [183,184,185]. Mutation of p53 can also lead to substantial immune dysfunction, resulting in the secretion of tumorigenic cytokines that support cell growth and cancer progression [186]. Missense mutations in p53 have recently been demonstrated to increase the expression of programmed cell death ligand 1 (PD-L1) and can therefore be utilized to predict treatment responses to the anti-PD-L1 immune checkpoint inhibitor, nivolumab [187,188,189,190].

An alternative approach for targeting p53 dysfunction that is of particular interest to our group is targeting p53 transcriptional activation [24,191]. Typically, p53 activation is thought of as an accumulation of p53 protein due to decreased turnover by MDM2, without activation of p53 transcription [30,50,51,59]. Unsurprisingly, p53 transcription is also induced by oncogenic stimuli. For example, DNA-damaging chemotherapeutic agents and ionizing radiation have been found to increase p53 transcription [43,44,47]. The DDR pathway activates Chk1 and Chk2 [48], both of which lead to the accumulation of p73 and E2F1 [192]. These two proteins can directly bind to the p53 promoter and facilitate p53 transcription [43,44]. p53 then negatively regulates DDR signaling by inducing the transcription of p21, which facilitates the downregulation of Chk1 (Figure 1A) [193].

**Figure 3:**
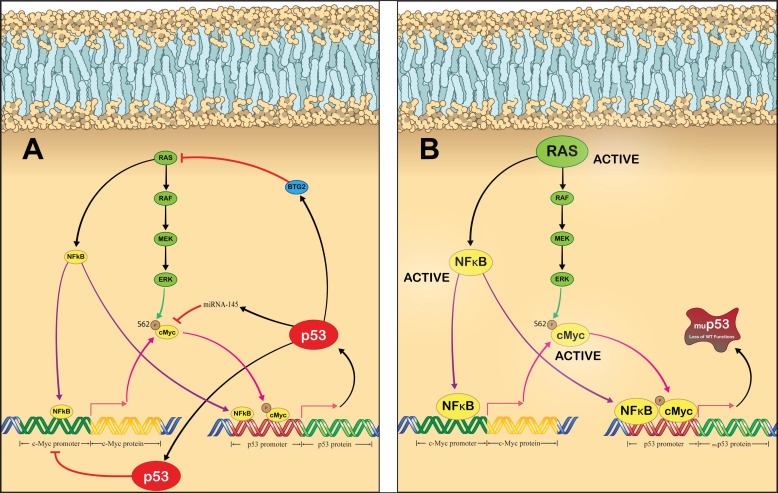
Mechanisms of p53 transcriptional activation and potential effects of p53 loss. Schemes follow another format. (**A**) Regulation of p53 expression in normal cells. RAS signaling leads to the activation of NF-κB as well as c-Myc, both of which directly bind to the p53 promoter and facilitate its expression. NF-kB also binds to the c-Myc promoter and induces its expression. The p53 protein then regulates these pathways, by stimulating the production of BTG2 and miRNA-145, which inhibit RAS and c-Myc function, respectively. Additionally, p53 protein can directly bind to the c-Myc promoter and repress its transcription. (**B**) Proposed effect that p53 loss can have on p53 promoter activity. Loss of p53 function can result in persistent RAS signaling through NF-κB and c-Myc, both of which will directly bind to and stimulate the p53 promoter. In cells with constitutively active mutant RAS and/or c-Myc, this pathway may be active even in the absence of p53 mutation/silencing. Legend: Arrows represent activation pathways (purple for NF-κB, pink for c-Myc, black for RAS and p53, green for phosphorylation event by ERK) and hammers represent inhibition.

Various oncogenes have been demonstrated to directly bind to the p53 promoter and facilitate its expression, for example, c-Myc [80] and NF-κB [107]. In most cases, p53 acts as a negative regulator of these oncogenes, inhibiting their activity as well as their transcriptional activation of p53 following induction [194,195]. As p53 is a master regulator of its own transcription, it is enticing to speculate about the impact that a loss of p53 function would have on the transcriptional activation state of p53. Perhaps one of the most interesting possibilities is cooperative regulation by c-Myc and RAS; both of which are frequently mutated and activated in many cancers [82,196,197]. RAS and c-Myc are intricately linked, with aberrant RAS signaling resulting in increased c-Myc protein stability [198]. c-Myc directly binds to and positively regulates the p53 promoter [80]. Conversely, p53 can bind to the c-Myc promoter and inhibit its expression [199]. Additionally, the p53 target, miR-145, can silence c-Myc [194]. It is possible that a loss of p53 can directly increase c-Myc activity, therefore resulting in upregulation of p53 promoter activity. This has been partially demonstrated before, as p53 loss was found to increase c-Myc expression and the expression of c-Myc regulated genes in breast cancer stem cells [200]. RAS and p53 also share a link, but it is far less direct than connection between p53 and c-Myc. Wild-type p53 has been demonstrated to facilitate expression of B-cell translocation gene 2 (BTG2), which functions as an inhibitor of aberrantly activate RAS [201]. Therefore, a loss of p53 could result in BTG2 loss, which causes RAS activity to increase, leading to increased stability of c-Myc and transcriptional activation of c-Myc regulated genes, including p53. RAS has also been demonstrated to activate NF-κB, which in addition to increasing c-Myc expression [198] also directly binds to the p53 promoter and induces its expression (Figure 3A). Oncogenic signaling through these proteins would remain active in the event of p53 loss, resulting in persistent activation of the p53 promoter. Additionally, p53 loss would mean that the cell loses the ability to halt the DDR pathway, causing p73 and E2F1 to activate the p53 promoter as well. We hypothesize that loss of p53 in cancer cells would lead to a non-productive upregulation of p53 transcription due to a loss of p53-mediated negative feedback (Figure 3B). The examples above were just a handful of potential networks that can become impaired following p53 loss [35,202]. The challenge then becomes developing a treatment strategy that can capitalize on this dysfunctional transcription network and generate meaningful clinical responses in patients. With the recent successes of novel genetic medicines, we may soon be able to modulate p53 signaling at a transcriptional level.

## 7. Future Directions

Rather than targeting specific aspects of p53 inactivation, we propose taking a multifaceted approach to target the p53 network. The Hsp90 inhibitors mentioned above give a great example of this. SAHA and ganetespib facilitate degradation of mup53 protein that abrogates tumorigenic potential in animal models of cancer but failed to perform in the clinic [164,165,166,167]. When combined with MDM2 and MDMX inhibitors, the efficacy of treatment was enhanced [149,150], indicating that combining modalities may offer some treatment benefits as long as there is no overlapping toxicity (Table 1). Therefore, it may be possible to improve treatment responses by combining these Hsp90 inhibitors with therapeutics such as Ad-p53 [113,114,115], which would activate WT p53 signaling while simultaneously blocking the effects of mup53—preventing a dominant negative effect from occurring—while also thwarting GOF mup53 signaling pathways [122]. Of course, this is purely speculative and proper experimentation is required to determine the feasibility of multimodal p53 targeting.

An alternative strategy for targeting the p53 network relies on the use of gene therapy. The WT p53 adenovirus expression vectors have arguably demonstrated the most clinical success of any p53 targeted therapy thus far, but they still face many limitations, primarily low treatment responses and cancer cell adaptation to the WT protein [203]. An additional limitation relates to the use of viral vectors as a gene delivery platform. Adaptive immune responses to the viral vector itself prevents repeat administration and therefore also long-term tumor control [204,205,206,207,208,209,210]. Non-viral gene delivery platforms, such as lipid nanoparticles, represent a non-immunogenic alternative to viral platforms and have demonstrated remarkable success in the delivery of nucleic acids [211,212,213,214,215]. Given the poor clinical responses to current p53 targeting approaches, it is reasonable to assume that tumors can overcome restoration of p53 activity. Therefore, it is necessary to examine the impact that p53 loss can have on the entire p53 network. Because p53 has such an integral role in regulating its own expression, loss of p53 function may result in an upregulation in the activity of the p53 promoter due to loss of negative feedback (Figure 3B) [35,202]. A simple approach to capitalize on the transcriptional dysfunction of the p53 network would be to design a gene therapy vector where the expression of a therapeutic protein is controlled by the p53 promoter and deliver this vector using a non-viral nucleic acid delivery platform [191]. The encoded therapeutic protein could directly target tumor cells, or indirectly target tumor cells by targeting the immune system or the cell cycle. Direct tumor-targeting therapies using proapoptotic proteins (Bax, NOXA, PIG3, APAF1) [216] or tumor suppressors (miR-7, 15-PGDH) are in preclinical or clinical studies [217]. Research into targeting the immune system using immunogenic proteins (cytokines, co-stimulatory proteins, tumor neoantigens) as a therapy for solid tumor cancers has rapidly increased recently which has translated into significant progress in the field of cancer vaccines [218]. Targeting the cell cycle using cytostatic proteins also shows promise as a cancer therapy strategy since tumor cells show aberrant activity from numerous cell cycle proteins [219]. Although for the treatment of solid tumor cancers, therapies that utilize a combination of these therapeutic proteins may be the most effective strategy due to synergistic anticancer activity, overcoming clonal heterogeneity, reducing regimen toxicity and drug resistance, and removing adaptive resistance by the tumor cells [220]. Combination therapies also allow treatments to be modified to maximize clinical responses.

## 8. Conclusions

Despite the complexities of p53 signaling, precision treatment using p53 remains a rapidly growing field in cancer therapy because so many aspects of tumorigenesis are influenced by p53. Unfortunately, despite promising preclinical data, most therapeutic strategies directed toward the modulation of p53 signaling have had low to moderate clinical success. There are a few compounds that show promise though, such as SAHA and ganetespib (Table 1), that were examined without considering p53 status in the inclusion criteria, and therefore treatment responses can likely be improved [166,167]. As we understand how each form of p53 inactivation impacts tumor biology, our ability to identify patient populations that can benefit from treatment grows larger. Precision medicine is undoubtedly the future of cancer treatment [221,222,223], and as such, molecular targets that function in nearly all subtypes of cancer will certainly carve out an essential role in this new era of treatment.

## Figures and Tables

**Table 1:** Recent clinical trials.

MDM2 Inhibitors			
Compound	Indication	Phase	Clinical Trial Identifier
RG7112	Hematological neoplasms	I	NCT00623870
	Acute Myeloid Leukemia	Ib/II	NCT03850535
RG7388	Acute Myeloid Leukemia	III	NCT02545283
	Solid Tumors	I	NCT00559533
	Multiple Myeloma	I/II	NCT02633059
	Follicular Lymphoma and Large B-Cell Lymphoma	Ib/II	NCT03135262
	Non-Hodgkin’s Lymphoma	I/II	NCT02624986
	Acute Myeloid Leukemia	I	NCT02670044
	Breast Cancer (Stage IV, Estrogen Receptor +)	I/II	NCT03566485
MK-8242	Acute Myeloid Leukemia	I	NCT01451437
	Solid Tumors	I	NCT01463696
AMG-232	Solid Tumors or Multiple Myeloma	I	NCT01723020
	Acute Myeloid Leukemia	I	NCT02016729
	Metastatic Melanoma	Ib/IIa	NCT02110355
	Multiple Myeloma	I	NCT03031730
	Soft Tissue Sarcoma	Ib	NCT03217266
	Acute Myeloid Leukemia	Ib	NCT04190550
**MDM2/MDMX Inhibitor**			
ALRN-6924	Hematological neoplasms and Small Tumors	I	NCT03654716
	Metastatic Solid Tumors	I	NCT03725436
	Solid Tumors or Lymphoma	I/IIa	NCT02264613
**Mutant p53 Reactivators**			
APR-246	Acute Myeloid Leukemia or Myelodysplastic Syndromes	II	NCT03931291
	Myeloid Malignancy	I	NCT04214860
	Solid Tumors	I/II	NCT04383938
	Esophageal Cancer	Ib/II	NCT02999893
	Non-Hodgkin’s Lymphoma, Mantle Cell Lymphoma, Chronic Lymphocytic Leukemia	I/II	NCT04419389
	Myeloid Malignancy	Ib/II	NCT03588078
	Myelodysplastic Syndromes	III	NCT03745716
	High Grade Serous Ovarian Cancer	II	NCT03268382
	Melanoma (BRAF/V600)	Ib/II	NCT03391050
	Myeloid Malignancy	Ib/II	NCT03072043
	High Grade Serous Ovarian Cancer	Ib/II	NCT02098343
	Hematological neoplasms and Prostate Cancer	I	NCT00900614
Ganetespib	Metastatic Ovarian Cancer (Mutant p53)	I/II	NCT02012192
SAHA	Advanced Cancer (Mutant p53)	I	NCT02042989
	Metastatic Melanoma	II	NCT00121225
	Advanced Cancer	I	NCT00324480
**p53 Gene Therapy**			
Ad-p53 + Immune Checkpoint Inhibitor	Solid Tumors or Lymphoma	II	NCT03544723
	Head and Neck Cancer or Metastatic Solid Tumors	I/II	NCT02842125

## References

[1] Bray F., Laversanne M., Weiderpass E., Soerjomataram I. (2021). The Ever-Increasing Importance of Cancer as a Leading Cause of Premature Death Worldwide. Cancer.

[2] Sung H., Ferlay J., Siegel R.L., Laversanne M., Soerjomataram I., Jemal A., Bray F. (2021). Global Cancer Statistics 2020: GLOBOCAN Estimates of Incidence and Mortality Worldwide for 36 Cancers in 185 Countries. CA Cancer J. Clin..

[3] Knudson A.G. (1971). Mutation and Cancer: Statistical Study of Retinoblastoma. Proc. Natl. Acad. Sci. USA.

[4] Kennedy S.R., Loeb L.A., Herr A.J. (2012). Somatic Mutations in Aging, Cancer and Neurodegeneration. Mech. Ageing Dev..

[5] Stoletov K., Beatty P.H., Lewis J.D. (2020). Novel Therapeutic Targets for Cancer Metastasis. Expert Rev. Anticancer Ther..

[6] Rudzinski J.K., Govindasamy N.P., Lewis J.D., Jurasz P. (2020). The Role of the Androgen Receptor in Prostate Cancer-Induced Platelet Aggregation and Platelet-Induced Invasion. J. Thromb. Haemost..

[7] Hanahan D., Weinberg R.A. (2000). The Hallmarks of Cancer. Cell.

[8] Hanahan D., Weinberg R.A. (2011). Hallmarks of Cancer: The Next Generation. Cell.

[9] Li X., Buckley B., Stoletov K., Jing Y., Ranson M., Lewis J.D., Kelso M., Fliegel L. (2021). Roles of the Na+/H+ Exchanger Isoform 1 and Urokinase in Prostate Cancer Cell Migration and Invasion. Int. J. Mol. Sci..

[10] Abou-Ouf H., Assem H., Ghosh S., Karnes R.J., Stoletov K., Palanisamy N., Lewis J.D., Bismar T.A. (2021). High Serine-Arginine Protein Kinase 1 Expression with PTEN Loss Defines Aggressive Phenotype of Prostate Cancer Associated with Lethal Outcome and Decreased Overall Survival. Eur. Urol. Open Sci..

[11] Yankaskas C.L., Bera K., Stoletov K., Serra S.A., Carrillo-Garcia J., Tuntithavornwat S., Mistriotis P., Lewis J.D., Valverde M.A., Konstantopoulos K. (2021). The Fluid Shear Stress Sensor TRPM7 Regulates Tumor Cell Intravasation. Sci. Adv..

[12] Kanwar N., Carmine-Simmen K., Nair R., Wang C., Moghadas-Jafari S., Blaser H., Tran-Thanh D., Wang D., Wang P., Wang J. (2020). Amplification of a Calcium Channel Subunit CACNG4 Increases Breast Cancer Metastasis. eBioMedicine.

[13] Fortunato A., Boddy A., Mallo D., Aktipis A., Maley C.C., Pepper J.W. (2017). Natural Selection in Cancer Biology: From Molecular Snowflakes to Trait Hallmarks. Cold Spring Harb. Perspect. Med..

[14] Shah S.P., Morin R.D., Khattra J., Prentice L., Pugh T., Burleigh A., Delaney A., Gelmon K., Guliany R., Senz J. (2009). Mutational Evolution in a Lobular Breast Tumour Profiled at Single Nucleotide Resolution. Nature.

[15] Mansoori B., Mohammadi A., Davudian S., Shirjang S., Baradaran B. (2017). The Different Mechanisms of Cancer Drug Resistance: A Brief Review. Adv. Pharm. Bull..

[16] Shaw A.T., Friboulet L., Leshchiner I., Gainor J.F., Bergqvist S., Brooun A., Burke B.J., Deng Y.-L., Liu W., Dardaei L. (2016). Resensitization to Crizotinib by the Lorlatinib ALK Resistance Mutation L1198F. N. Engl. J. Med..

[17] Alfarouk K.O., Stock C.-M., Taylor S., Walsh M., Muddathir A.K., Verduzco D., Bashir A.H.H., Mohammed O.Y., Elhassan G.O., Harguindey S. (2015). Resistance to Cancer Chemotherapy: Failure in Drug Response from ADME to P-Gp. Cancer Cell Int..

[18] Croker A.K., Rodriguez-Torres M., Xia Y., Pardhan S., Leong H.S., Lewis J.D., Allan A.L. (2017). Differential Functional Roles of ALDH1A1 and ALDH1A3 in Mediating Metastatic Behavior and Therapy Resistance of Human Breast Cancer Cells. Int. J. Mol. Sci..

[19] Zugazagoitia J., Guedes C., Ponce S., Ferrer I., Molina-Pinelo S., Paz-Ares L. (2016). Current Challenges in Cancer Treatment. Clin. Ther..

[20] Arnedos M., Soria J.-C., Andre F., Tursz T. (2014). Personalized Treatments of Cancer Patients: A Reality in Daily Practice, a Costly Dream or a Shared Vision of the Future from the Oncology Community?. Cancer Treat. Rev..

[21] Bell C.J., Potts K.G., Hitt M.M., Pink D., Tuszynski J.A., Lewis J.D. (2022). Novel Colchicine Derivative CR42-24 Demonstrates Potent Anti-Tumor Activity in Urothelial Carcinoma. Cancer Lett..

[22] Paproski R.J., Jovel J., Wong G.K.S., Lewis J.D., Zemp R.J. (2017). Enhanced Detection of Cancer Biomarkers in Blood-Borne Extracellular Vesicles Using Nanodroplets and Focused Ultrasound. Cancer Res..

[23] Stoletov K., Willetts L., Beatty P.H., Lewis J.D. (2021). Discovery of Metastatic Regulators Using a Rapid and Quantitative Intravital Chick Chorioallantoic Membrane Model. J. Vis. Exp..

[24] Wang B., Wu H., Chai C., Lewis J., Pichiorri F., Eisenstat D.D., Pomeroy S.L., Leng R.P. (2017). MicroRNA-1301 Suppresses Tumor Cell Migration and Invasion by Targeting the p53/UBE4B Pathway in Multiple Human Cancer Cells. Cancer Lett..

[25] Brown D.W., Wee P., Bhandari P., Vega H., Grin L., Sosnowski D., Hejazi M., Ablack J., Clancy E.K., Pink D. (2022). Safe and Effective Delivery of Nucleic Acids Using Proteolipid Vehicles Formulated with Fusion-Associated Small Transmembrane Proteins. SSRN Preprint. https://ssrn.com/abstract=4241169.

[26] Cooper T.T., Dieters-Castator D.Z., Liu J., Siegers G.M., Pink D., Lewis J.D., Fu Y., Steed H., Lajoie G.A., Postovit L.-M. (2022). Plasma EV Biomarkers of High-Grade Serous Carcinoma Targeted Proteomics and Support Vector Classification Reveal Potential Biomarkers for the Early Detection of High-Grade Serous Ovarian Cancer. Ph.D. Thesis.

[27] Kedarisetti P., Bouvet V.R., Shi W., Bergman C.N., Dufour J., Kashani Ilkhechi A., Bell K.L., Paproski R.J., Lewis J.D., Wuest F.R. (2020). Enrichment and Ratiometric Detection of Circulating Tumor Cells Using PSMA- and Folate Receptor-Targeted Magnetic and Surface-Enhanced Raman Scattering Nanoparticles. Biomed. Opt. Express.

[28] Lane D.P. (1992). Cancer. p53, Guardian of the Genome. Nature.

[29] Vogelstein B., Lane D., Levine A.J. (2000). Surfing the p53 Network. Nature.

[30] Bieging K.T., Mello S.S., Attardi L.D. (2014). Unravelling Mechanisms of p53-Mediated Tumour Suppression. Nat. Rev. Cancer.

[31] Freed-Pastor W.A., Prives C. (2012). Mutant p53: One Name, Many Proteins. Genes Dev..

[32] Stegh A.H. (2012). Targeting the p53 Signaling Pathway in Cancer Therapy—The Promises, Challenges and Perils. Expert Opin. Ther. Targets.

[33] Vousden K.H., Prives C. (2009). Blinded by the Light: The Growing Complexity of p53. Cell.

[34] Saldana-Meyer R., Recillas-Targa F. (2011). Transcriptional and Epigenetic Regulation of the p53 Tumor Suppressor Gene. Epigenetics.

[35] Harris S.L., Levine A.J. (2005). The p53 Pathway: Positive and Negative Feedback Loops. Oncogene.

[36] Oren M. (2003). Decision Making by p53: Life, Death and Cancer. Cell Death Differ..

[37] Lin T., Hou P.F., Meng S., Chen F., Jiang T., Li M.L., Shi M.L., Liu J.J., Zheng J.N., Bai J. (2019). Emerging Roles of p53 Related LncRNAs in Cancer Progression: A Systematic Review. Int. J. Biol. Sci..

[38] Haupt Y., Maya R., Kazaz A., Oren M. (1997). Mdm2 Promotes the Rapid Degradation of p53. Nature.

[39] Kubbutat M.H., Jones S.N., Vousden K.H. (1997). Regulation of p53 Stability by Mdm2. Nature.

[40] Shvarts A., Steegenga W.T., Riteco N., van Laar T., Dekker P., Bazuine M., van Ham R.C., van der Houven van Oordt W., Hateboer G., van der Eb A.J. (1996). MDMX: A Novel p53-Binding Protein with Some Functional Properties of MDM2. EMBO J..

[41] Jackson M.W., Berberich S.J. (2000). MdmX Protects p53 from Mdm2-Mediated Degradation. Mol. Cell Biol..

[42] Wade M., Wahl G.M. (2009). Targeting Mdm2 and Mdmx in Cancer Therapy: Better Living through Medicinal Chemistry?. Mol. Cancer Res..

[43] Wang S., El-Deiry W.S. (2006). P73 or p53 Directly Regulates Human p53 Transcription to Maintain Cell Cycle Checkpoints. Cancer Res..

[44] Bug M., Dobbelstein M. (2011). Anthracyclines Induce the Accumulation of Mutant p53 through E2F1-Dependent and -Independent Mechanisms. Oncogene.

[45] Kogan-Sakin I., Tabach Y., Buganim Y., Molchadsky A., Solomon H., Madar S., Kamer I., Stambolsky P., Shelly A., Goldfinger N. (2011). Mutant p53(R175H) Upregulates Twist1 Expression and Promotes Epithelial-Mesenchymal Transition in Immortalized Prostate Cells. Cell Death Differ..

[46] Fei P., El-Deiry W.S. (2003). p53 and Radiation Responses. Oncogene.

[47] Shirley S.H., Rundhaug J.E., Tian J., Cullinan-Ammann N., Lambertz I., Conti C.J., Fuchs-Young R. (2009). Transcriptional Regulation of Estrogen Receptor-α by p53 in Human Breast Cancer Cells. Cancer Res..

[48] Bartek J., Lukas J. (2003). Chk1 and Chk2 Kinases in Checkpoint Control and Cancer. Cancer Cell.

[49] Maya R., Balass M., Kim S.T., Shkedy D., Martinez Leal J.F., Shifman O., Moas M., Buschmann T., Ronai Z., Shiloh Y. (2001). ATM-Dependent Phosphorylation of Mdm2 on Serine 395: Role in p53 Activation by DNA Damage. Genes Dev..

[50] Shieh S.Y., Ikeda M., Taya Y., Prives C. (1997). DNA Damage-Induced Phosphorylation of p53 Alleviates Inhibition by MDM2. Cell.

[51] Chehab N.H., Malikzay A., Stavridi E.S., Halazonetis T.D. (1999). Phosphorylation of Ser-20 Mediates Stabilization of Human p53 in Response to DNA Damage. Proc. Natl. Acad. Sci. USA.

[52] Tibbetts R.S., Brumbaugh K.M., Williams J.M., Sarkaria J.N., Cliby W.A., Shieh S.Y., Taya Y., Prives C., Abraham R.T. (1999). A Role for ATR in the DNA Damage-Induced Phosphorylation of p53. Genes Dev..

[53] Pereg Y., Shkedy D., de Graaf P., Meulmeester E., Edelson-Averbukh M., Salek M., Biton S., Teunisse A.F.A.S., Lehmann W.D., Jochemsen A.G. (2005). Phosphorylation of Hdmx Mediates Its Hdm2- and ATM-Dependent Degradation in Response to DNA Damage. Proc. Natl. Acad. Sci. USA.

[54] Kawai H., Wiederschain D., Kitao H., Stuart J., Tsai K.K.C., Yuan Z.-M. (2003). DNA Damage-Induced MDMX Degradation Is Mediated by MDM2*. J. Biol. Chem..

[55] Jin Y., Dai M.-S., Lu S.Z., Xu Y., Luo Z., Zhao Y., Lu H. (2006). 14-3-3γ Binds to MDMX That Is Phosphorylated by UV-Activated Chk1, Resulting in p53 Activation. EMBO J..

[56] Barak Y., Juven T., Haffner R., Oren M. (1993). Mdm2 Expression Is Induced by Wild Type p53 Activity. EMBO J..

[57] Lu X., Ma O., Nguyen T.A., Jones S.N., Oren M., Donehower L.A. (2007). The Wip1 Phosphatase Acts as a Gatekeeper in the p53-Mdm2 Autoregulatory Loop. Cancer Cell.

[58] Komori H., Enomoto M., Nakamura M., Iwanaga R., Ohtani K. (2005). Distinct E2F-Mediated Transcriptional Program Regulates P14ARF Gene Expression. EMBO J..

[59] Pomerantz J., Schreiber-Agus N., Liégeois N.J., Silverman A., Alland L., Chin L., Potes J., Chen K., Orlow I., Lee H.-W. (1998). The Ink4a Tumor Suppressor Gene Product, P19ARF, Interacts with MDM2 and Neutralizes MDM2′s Inhibition of p53. Cell.

[60] Fogal V., Hsieh J.-K., Royer C., Zhong S., Lu X. (2005). Cell Cycle-Dependent Nuclear Retention of p53 by E2F1 Requires Phosphorylation of p53 at Ser315. EMBO J..

[61] Gartel A.L., Radhakrishnan S.K. (2005). Lost in Transcription: P21 Repression, Mechanisms, and Consequences. Cancer Res..

[62] Gencel-Augusto J., Lozano G. (2020). REVIEW p53 Tetramerization: At the Center of the Dominant-Negative Effect of Mutant p53. Genes Dev..

[63] Fischer M. (2017). Census and Evaluation of p53 Target Genes. Oncogene.

[64] Kracikova M., Akiri G., George A., Sachidanandam R., Aaronson S.A. (2013). A Threshold Mechanism Mediates p53 Cell Fate Decision between Growth Arrest and Apoptosis. Cell Death Differ..

[65] Zhang X.-P., Liu F., Wang W. (2011). Two-Phase Dynamics of p53 in the DNA Damage Response. Proc. Natl. Acad. Sci. USA.

[66] Williams A.B., Schumacher B. (2016). p53 in the DNA-Damage-Repair Process. Cold Spring Harb. Perspect. Med..

[67] Mijit M., Caracciolo V., Melillo A., Amicarelli F., Giordano A. (2020). Role of p53 in the Regulation of Cellular Senescence. Biomolecules.

[68] Yosef R., Pilpel N., Papismadov N., Gal H., Ovadya Y., Vadai E., Miller S., Porat Z., Ben-Dor S., Krizhanovsky V. (2017). P21 Maintains Senescent Cell Viability under Persistent DNA Damage Response by Restraining JNK and Caspase Signaling. EMBO J..

[69] Gupta S., Silveira D.A., Mombach J.C.M. (2020). Towards DNA-Damage Induced Autophagy: A Boolean Model of p53-Induced Cell Fate Mechanisms. DNA Repair.

[70] Yun C.W., Lee S.H. (2018). The Roles of Autophagy in Cancer. Int. J. Mol. Sci..

[71] Mrakovcic M., Fröhlich L.F. (2018). p53-Mediated Molecular Control of Autophagy in Tumor Cells. Biomolecules.

[72] Crighton D., Wilkinson S., O’Prey J., Syed N., Smith P., Harrison P.R., Gasco M., Garrone O., Crook T., Ryan K.M. (2006). DRAM, a p53-Induced Modulator of Autophagy, Is Critical for Apoptosis. Cell.

[73] Kenzelmann Broz D., Mello S.S., Bieging K.T., Jiang D., Dusek R.L., Brady C.A., Sidow A., Attardi L.D. (2013). Global Genomic Profiling Reveals an Extensive p53-Regulated Autophagy Program Contributing to Key p53 Responses. Genes Dev..

[74] White E. (2016). Autophagy and p53. Cold Spring Harb. Perspect. Med..

[75] Mercer W.E., Shields M.T., Amin M., Sauve G.J., Appella E., Romano J.W., Ullrich S.J. (1990). Negative Growth Regulation in a Glioblastoma Tumor Cell Line That Conditionally Expresses Human Wild-Type p53. Proc. Natl. Acad. Sci. USA.

[76] Baker S.J., Markowitz S., Fearon E.R., Willson J.K., Vogelstein B. (1990). Suppression of Human Colorectal Carcinoma Cell Growth by Wild-Type p53. Science.

[77] Donehower L.A., Harvey M., Slagle B.L., McArthur M.J., Montgomery C.A., Butel J.S., Bradley A. (1992). Mice Deficient for p53 Are Developmentally Normal but Susceptible to Spontaneous Tumours. Nature.

[78] Nigro J.M., Baker S.J., Preisinger A.C., Jessup J.M., Hosteller R., Cleary K., Signer S.H., Davidson N., Baylin S., Devilee P. (1989). Mutations in the p53 Gene Occur in Diverse Human Tumour Types. Nature.

[79] Baker S.J., Fearon E.R., Nigro J.M., Hamilton S.R., Preisinger A.C., Jessup J.M., vanTuinen P., Ledbetter D.H., Barker D.F., Nakamura Y. (1989). Chromosome 17 Deletions and p53 Gene Mutations in Colorectal Carcinomas. Science.

[80] Roy B., Beamon J., Balint E., Reisman D. (1994). Transactivation of the Human p53 Tumor Suppressor Gene by C-Myc/Max Contributes to Elevated Mutant p53 Expression in Some Tumors. Mol. Cell Biol..

[81] Raman V., Martensen S.A., Reisman D., Evron E., Odenwald W.F., Jaffee E., Marks J., Sukumar S. (2000). Compromised HOXA5 Function Can Limit p53 Expression in Human Breast Tumours. Nature.

[82] Gabay M., Li Y., Felsher D.W. (2014). MYC Activation Is a Hallmark of Cancer Initiation and Maintenance. Cold Spring Harb. Perspect. Med..

[83] Ordonez-Moran P., Dafflon C., Imajo M., Nishida E., Huelsken J. (2015). HOXA5 Counteracts Stem Cell Traits by Inhibiting Wnt Signaling in Colorectal Cancer. Cancer Cell.

[84] Teo W.W., Merino V.F., Cho S., Korangath P., Liang X., Wu R.C., Neumann N.M., Ewald A.J., Sukumar S. (2016). HOXA5 Determines Cell Fate Transition and Impedes Tumor Initiation and Progression in Breast Cancer through Regulation of E-Cadherin and CD24. Oncogene.

[85] Wilson J.R.F., Bateman A.C., Hanson H., An Q., Evans G., Rahman N., Jones J.L., Eccles D.M. (2010). A Novel HER2-Positive Breast Cancer Phenotype Arising from Germline TP53 Mutations. J. Med. Genet..

[86] Eastham J.A., Stapleton A.M., Gousse A.E., Timme T.L., Yang G., Slawin K.M., Wheeler T.M., Scardino P.T., Thompson T.C. (1995). Association of p53 Mutations with Metastatic Prostate Cancer. Clin. Cancer Res..

[87] Grignon D.J., Sarkar F.H., Forman J.D., Caplan R., Pajak T.F., Lawton C.A., Hammond E.H., Pilepich M.V., Mesic J., Fu K.K. (1997). p53 Status and Prognosis of Locally Advanced Prostatic Adenocarcinoma: A Study Based on RTOG 8610. JNCI J. Natl. Cancer Inst..

[88] Liu J., Zhang C., Feng Z. (2014). Tumor Suppressor p53 and Its Gain-of-Function Mutants in Cancer. Acta Biochim. Biophys. Sin..

[89] Baliou E., Nonni A., Keramopoulos D., Ragos V., Tsiambas E., Patsouris E., Pavlakis K. (2016). Deregulation of p53-MDM2 Auto-Regulatory Pathway in Breast Carcinoma. J. BUON.

[90] Oren M., Rotter V. (2010). Mutant p53 Gain-of-Function in Cancer. Cold Spring Harb. Perspect. Biol..

[91] Mello S.S., Attardi L.D. (2013). Not All p53 Gain-of-Function Mutants Are Created Equal. Cell Death Differ..

[92] Gaiddon C., Lokshin M., Ahn J., Zhang T., Prives C. (2001). A Subset of Tumor-Derived Mutant Forms of p53 down-Regulate P63 and P73 through a Direct Interaction with the p53 Core Domain. Mol. Cell Biol..

[93] Dötsch V., Bernassola F., Coutandin D., Candi E., Melino G. (2010). P63 and P73, the Ancestors of p53. Cold Spring Harb. Perspect. Biol..

[94] Liu G., McDonnell T.J., Montes de Oca Luna R., Kapoor M., Mims B., El-Naggar A.K., Lozano G. (2000). Solo, MHigh Metastatic Potential in Mice Inheriting a Targeted p53 Missense Mutation. Proc. Natl. Acad. Sci. USA.

[95] Olive K.P., Tuveson D.A., Ruhe Z.C., Yin B., Willis N.A., Bronson R.T., Crowley D., Jacks T. (2004). Mutant p53 Gain of Function in Two Mouse Models of Li-Fraumeni Syndrome. Cell.

[96] Lang G.A., Iwakuma T., Suh Y.A., Liu G., Rao V.A., Parant J.M., Valentin-Vega Y.A., Terzian T., Caldwell L.C., Strong L.C. (2004). Gain of Function of a p53 Hot Spot Mutation in a Mouse Model of Li-Fraumeni Syndrome. Cell.

[97] Solomon H., Dinowitz N., Pateras I.S., Cooks T., Shetzer Y., Molchadsky A., Charni M., Rabani S., Koifman G., Tarcic O. (2018). Mutant p53 Gain of Function Underlies High Expression Levels of Colorectal Cancer Stem Cells Markers. Oncogene.

[98] Roman-Rosales A.A., Garcia-Villa E., Herrera L.A., Gariglio P., Diaz-Chavez J. (2018). Mutant p53 Gain of Function Induces HER2 Over-Expression in Cancer Cells. BMC Cancer.

[99] Schwartzenberg-Bar-Yoseph F., Armoni M., Karnieli E. (2004). The Tumor Suppressor p53 Down-Regulates Glucose Transporters GLUT1and GLUT4 Gene Expression. Cancer Res.

[100] Bensaad K., Tsuruta A., Selak M.A., Vidal M.N.C., Nakano K., Bartrons R., Gottlieb E., Vousden K.H. (2006). TIGAR, a p53-Inducible Regulator of Glycolysis and Apoptosis. Cell.

[101] Chang C.-J., Chao C.-H., Xia W., Yang J.-Y., Xiong Y., Li C.-W., Yu W.-H., Rehman S.K., Hsu J.L., Lee H.-H. (2011). p53 Regulates Epithelial–Mesenchymal Transition and Stem Cell Properties through Modulating MiRNAs. Nat. Cell Biol..

[102] Ren D., Wang M., Guo W., Zhao X., Tu X., Huang S., Zou X., Peng X. (2013). Wild-Type p53 Suppresses the Epithelial-Mesenchymal Transition and Stemness in PC-3 Prostate Cancer Cells by Modulating MiR-145. Int. J. Oncol..

[103] Brighenti E., Calabrese C., Liguori G., Giannone F.A., Trerè D., Montanaro L., Derenzini M. (2014). Interleukin 6 Downregulates p53 Expression and Activity by Stimulating Ribosome Biogenesis: A New Pathway Connecting Inflammation to Cancer. Oncogene.

[104] Pastor D.M., Irby R.B., Poritz L.S. (2010). Tumor Necrosis Factor α Induces p53 Up-Regulated Modulator of Apoptosis Expression in Colorectal Cancer Cell Lines. Dis. Colon Rectum.

[105] Liu T., Zhang L., Joo D., Sun S.-C. (2017). NF-ΚB Signaling in Inflammation. Signal Transduct. Target Ther..

[106] Lu T., Burdelya L.G., Swiatkowski S.M., Boiko A.D., Howe P.H., Stark G.R., Gudkov A. (2004). V Secreted Transforming Growth Factor Beta2 Activates NF-KappaB, Blocks Apoptosis, and Is Essential for the Survival of Some Tumor Cells. Proc. Natl. Acad. Sci. USA.

[107] Kirch H.C., Flaswinkel S., Rumpf H., Brockmann D., Esche H. (1999). Expression of Human p53 Requires Synergistic Activation of Transcription from the p53 Promoter by AP-1, NF-KappaB and Myc/Max. Oncogene.

[108] Fujiwara T., Grimm E.A., Mukhopadhyay T., Cai D.W., Owen-Schaub L.B., Roth J.A. (1993). A Retroviral Wild-Type p53 Expression Vector Penetrates Human Lung Cancer Spheroids and Inhibits Growth by Inducing Apoptosis. Cancer Res..

[109] Shaw P., Bovey R., Tardy S., Sahli R., Sordat B., Costa J. (1992). Induction of Apoptosis by Wild-Type p53 in a Human Colon Tumor-Derived Cell Line. Proc. Natl. Acad. Sci. USA.

[110] Fujiwara T., Cai D.W., Georges R.N., Mukhopadhyay T., Grimm E.A., Roth J.A. (1994). Therapeutic Effect of a Retroviral Wild-Type p53 Expression Vector in an Orthotopic Lung Cancer Model. JNCI J. Natl. Cancer Inst..

[111] Roth J.A., Nguyen D., Lawrence D.D., Kemp B.L., Carrasco C.H., Ferson D.Z., Hong W.K., Komaki R., Lee J.J., Nesbitt J.C. (1996). Retrovirus–Mediated Wild–Type p53 Gene Transfer to Tumors of Patients with Lung Cancer. Nat. Med..

[112] Milone M.C., O’Doherty U. (2018). Clinical Use of Lentiviral Vectors. Leukemia.

[113] Zhang W.W., Fang X., Mazur W., French B.A., Georges R.N., Roth J.A. (1994). High-Efficiency Gene Transfer and High-Level Expression of Wild-Type p53 in Human Lung Cancer Cells Mediated by Recombinant Adenovirus. Cancer Gene Ther..

[114] Spitz F.R., Nguyen D., Skibber J.M., Meyn R.E., Cristiano R.J., Roth J.A. (1996). Adenoviral-Mediated Wild-Type p53 Gene Expression Sensitizes Colorectal Cancer Cells to Ionizing Radiation. Clin. Cancer Res..

[115] Nielsen L.L., Dell J., Maxwell E., Armstrong L., Maneval D., Catino J.J. (1997). Efficacy of p53 Adenovirus-Mediated Gene Therapy against Human Breast Cancer Xenografts. Cancer Gene Ther..

[116] Swisher S.G., Roth J.A., Nemunaitis J., Lawrence D.D., Kemp B.L., Carrasco C.H., Connors D.G., El-Naggar A.K., Fossella F., Glisson B.S. (1999). Adenovirus-Mediated p53 Gene Transfer in Advanced Non-Small-Cell Lung Cancer. JNCI J. Natl. Cancer Inst..

[117] Clayman G.L., el-Naggar A.K., Lippman S.M., Henderson Y.C., Frederick M., Merritt J.A., Zumstein L.A., Timmons T.M., Liu T.J., Ginsberg L. (1998). Adenovirus-Mediated p53 Gene Transfer in Patients with Advanced Recurrent Head and Neck Squamous Cell Carcinoma. J. Clin. Oncol..

[118] Peng Z., Han D., Zhang S., Pan J., Tang P., Xiao S., Chen C., Huang Z., Zhang W., Zhang X. (2003). Clinical Evaluation of Safety and Efficacy of Intratumoral Administration of a Recombinant Adenoviral-p53 Anticancer Agent (Genkaxin). Mol. Ther..

[119] Swisher S.G., Roth J.A., Komaki R., Gu J., Lee J.J., Hicks M., Ro J.Y., Hong W.K., Merritt J.A., Ahrar K. (2003). Induction of p53-Regulated Genes and Tumor Regression in Lung Cancer Patients after Intratumoral Delivery of Adenoviral p53 (INGN 201) and Radiation Therapy. Clin. Cancer Res..

[120] Zhang W.-W., Li L., Li D., Liu J., Li X., Li W., Xu X., Zhang M.J., Chandler L.A., Lin H. (2018). The First Approved Gene Therapy Product for Cancer Ad-p53 (Gendicine): 12 Years in the Clinic. Hum. Gene Ther..

[121] Chen G.-X., Zhang S., He X.-H., Liu S.-Y., Ma C., Zou X.-P. (2014). Clinical Utility of Recombinant Adenoviral Human p53 Gene Therapy: Current Perspectives. Onco Targets Ther..

[122] Wang Y., Suh Y.-A., Fuller M.Y., Jackson J.G., Xiong S., Terzian T., Quintás-Cardama A., Bankson J.A., El-Naggar A.K., Lozano G. (2011). Restoring Expression of Wild-Type p53 Suppresses Tumor Growth but Does Not Cause Tumor Regression in Mice with a p53 Missense Mutation. J. Clin. Investig..

[123] Monti P., Campomenosi P., Ciribilli Y., Iannone R., Inga A., Abbondandolo A., Resnick M.A., Fronza G. (2002). Tumour p53 Mutations Exhibit Promoter Selective Dominance over Wild Type p53. Oncogene.

[124] Clayman G.L., El-Naggar A.K., Roth J.A., Zhang W.-W., Goepfert H., Taylor D.L., Liu T.-J. (1995). In Vivo Molecular Therapy with p53 Adenovirus for Microscopic Residual Head and Neck Squamous Carcinoma. Cancer Res..

[125] Zhang W.-W., Alemany R., Wang J., Koch P.E., Ordonez N.G., Roth J.A. (1995). Safety Evaluation of Ad5CMY-p53 In Vitro and In Vivo. Hum. Gene Ther..

[126] Schirmbeck R., Reimann J., Kochanek S., Kreppel F. (2008). The Immunogenicity of Adenovirus Vectors Limits the Multispecificity of CD8 T-Cell Responses to Vector-Encoded Transgenic Antigens. Mol. Ther..

[127] Coughlan L. (2020). Factors Which Contribute to the Immunogenicity of Non-Replicating Adenoviral Vectored Vaccines. Front. Immunol..

[128] Tesniere A., Schlemmer F., Boige V., Kepp O., Martins I., Ghiringhelli F., Aymeric L., Michaud M., Apetoh L., Barault L. (2010). Immunogenic Death of Colon Cancer Cells Treated with Oxaliplatin. Oncogene.

[129] Casares N., Pequignot M.O., Tesniere A., Ghiringhelli F., Roux S., Chaput N., Schmitt E., Hamai A., Hervas-Stubbs S., Obeid M. (2005). Caspase-Dependent Immunogenicity of Doxorubicin-Induced Tumor Cell Death. J. Exp. Med..

[130] Sobol R.E., Menander K.B., Chada S., Wiederhold D., Sellman B., Talbott M., Nemunaitis J.J. (2021). Analysis of Adenoviral p53 Gene Therapy Clinical Trials in Recurrent Head and Neck Squamous Cell Carcinoma. Front. Oncol..

[131] Chada S., Wiederhold D., Menander K.B., Sellman B., Talbott M., Nemunaitis J.J., Ahn H.M., Jung B.K., Yun C.O., Sobol R.E. (2022). Tumor Suppressor Immune Gene Therapy to Reverse Immunotherapy Resistance. Cancer Gene Ther..

[132] Vassilev L.T., Vu B.T., Graves B., Carvajal D., Podlaski F., Filipovic Z., Kong N., Kammlott U., Lukacs C., Klein C. (2004). In Vivo Activation of the p53 Pathway by Small-Molecule Antagonists of MDM2. Science.

[133] Vu B., Wovkulich P., Pizzolato G., Lovey A., Ding Q., Jiang N., Liu J.-J., Zhao C., Glenn K., Wen Y. (2013). Discovery of RG7112: A Small-Molecule MDM2 Inhibitor in Clinical Development. ACS Med. Chem. Lett..

[134] Ding K., Lu Y., Nikolovska-Coleska Z., Qiu S., Ding Y., Gao W., Stuckey J., Krajewski K., Roller P.P., Tomita Y. (2005). Structure-Based Design of Potent Non-Peptide MDM2 Inhibitors. J. Am. Chem. Soc..

[135] Wang S., Sun W., Zhao Y., McEachern D., Meaux I., Barrière C., Stuckey J.A., Meagher J.L., Bai L., Liu L. (2014). SAR405838: An Optimized Inhibitor of MDM2–p53 Interaction That Induces Complete and Durable Tumor Regression. Cancer Res..

[136] Zhao Y., Liu L., Sun W., Lu J., McEachern D., Li X., Yu S., Bernard D., Ochsenbein P., Ferey V. (2013). Diastereomeric Spirooxindoles as Highly Potent and Efficacious MDM2 Inhibitors. J. Am. Chem. Soc..

[137] Sun D., Li Z., Rew Y., Gribble M., Bartberger M.D., Beck H.P., Canon J., Chen A., Chen X., Chow D. (2014). Discovery of AMG 232, a Potent, Selective, and Orally Bioavailable MDM2–p53 Inhibitor in Clinical Development. J. Med. Chem..

[138] Ray-Coquard I., Blay J.-Y., Italiano A., Le Cesne A., Penel N., Zhi J., Heil F., Rueger R., Graves B., Ding M. (2012). Effect of the MDM2 Antagonist RG7112 on the p53 Pathway in Patients with MDM2-Amplified, Well-Differentiated or Dedifferentiated Liposarcoma: An Exploratory Proof-of-Mechanism Study. Lancet Oncol..

[139] Mahfoudhi E., Lordier L., Marty C., Pan J., Roy A., Roy L., Rameau P., Abbes S., Debili N., Raslova H. (2016). p53 Activation Inhibits All Types of Hematopoietic Progenitors and All Stages of Megakaryopoiesis. Oncotarget.

[140] Haronikova L., Bonczek O., Zatloukalova P., Kokas-Zavadil F., Kucerikova M., Coates P.J., Fahraeus R., Vojtesek B. (2021). Resistance Mechanisms to Inhibitors of p53-MDM2 Interactions in Cancer Therapy: Can We Overcome Them?. Cell Mol. Biol. Lett..

[141] Wagner A.J., Banerji U., Mahipal A., Somaiah N., Hirsch H., Fancourt C., Johnson-Levonas A.O., Lam R., Meister A.K., Russo G. (2017). Phase I Trial of the Human Double Minute 2 Inhibitor MK-8242 in Patients with Advanced Solid Tumors. J. Clin. Oncol..

[142] Efeyan A., Ortega-Molina A., Velasco-Miguel S., Herranz D., Vassilev L.T., Serrano M. (2007). Induction of p53-Dependent Senescence by the MDM2 Antagonist Nutlin-3a in Mouse Cells of Fibroblast Origin. Cancer Res..

[143] Coppé J.P., Desprez P.Y., Krtolica A., Campisi J. (2010). The Senescence-Associated Secretory Phenotype: The Dark Side of Tumor Suppression. Annu. Rev. Pathol. Mech. Dis..

[144] Borthakur G., Duvvuri S., Ruvolo V., Tripathi D.N., Piya S., Burks J., Jacamo R., Kojima K., Ruvolo P., Fueyo-Margareto J. (2015). MDM2 Inhibitor, Nutlin 3a, Induces p53 Dependent Autophagy in Acute Leukemia by AMP Kinase Activation. PLoS ONE.

[145] Pechackova S., Burdova K., Benada J., Kleiblova P., Jenikova G., Macurek L. (2016). Inhibition of WIP1 Phosphatase Sensitizes Breast Cancer Cells to Genotoxic Stress and to MDM2 Antagonist Nutlin-3. Oncotarget.

[146] Puszynski K., Gandolfi A., d’Onofrio A. (2014). The Pharmacodynamics of the p53-Mdm2 Targeting Drug Nutlin: The Role of Gene-Switching Noise. PLoS Comput. Biol..

[147] Garcia D., Warr M.R., Martins C.P., Brown Swigart L., Passegué E., Evan G.I. (2011). Validation of MdmX as a Therapeutic Target for Reactivating p53 in Tumors. Genes Dev..

[148] Yu D.-H., Xu Z.-Y., Mo S., Yuan L., Cheng X.-D., Qin J.-J. (2020). Targeting MDMX for Cancer Therapy: Rationale, Strategies, and Challenges. Front. Oncol..

[149] Reed D., Shen Y., Shelat A.A., Arnold L.A., Ferreira A.M., Zhu F., Mills N., Smithson D.C., Regni C.A., Bashford D. (2010). Identification and Characterization of the First Small Molecule Inhibitor of MDMX. J. Biol. Chem..

[150] Karan G., Wang H., Chakrabarti A., Karan S., Liu Z., Xia Z., Gundluru M., Moreton S., Saunthararajah Y., Jackson M.W. (2016). Identification of a Small Molecule That Overcomes HdmX-Mediated Suppression of p53. Mol. Cancer Ther..

[151] Dewaele M., Tabaglio T., Willekens K., Bezzi M., Teo S.X., Low D.H.P., Koh C.M., Rambow F., Fiers M., Rogiers A. (2016). Antisense Oligonucleotide-Mediated MDM4 Exon 6 Skipping Impairs Tumor Growth. J. Clin. Investig..

[152] Bykov V.J.N., Issaeva N., Shilov A., Hultcrantz M., Pugacheva E., Chumakov P., Bergman J., Wiman K.G., Selivanova G. (2002). Restoration of the Tumor Suppressor Function to Mutant p53 by a Low-Molecular-Weight Compound. Nat. Med..

[153] Lambert J.M.R., Gorzov P., Veprintsev D.B., Söderqvist M., Segerbäck D., Bergman J., Fersht A.R., Hainaut P., Wiman K.G., Bykov V.J.N. (2009). PRIMA-1 Reactivates Mutant p53 by Covalent Binding to the Core Domain. Cancer Cell.

[154] Degtjarik O., Golovenko D., Diskin-Posner Y., Abrahmsén L., Rozenberg H., Shakked Z. (2021). Structural Basis of Reactivation of Oncogenic p53 Mutants by a Small Molecule: Methylene Quinuclidinone (MQ). Nat. Commun..

[155] Cluzeau T., Sebert M., Rahmé R., Cuzzubbo S., Lehmann-Che J., Madelaine I., Peterlin P., Bève B., Attalah H., Chermat F. (2021). Eprenetapopt Plus Azacitidine in TP53-Mutated Myelodysplastic Syndromes and Acute Myeloid Leukemia: A Phase II Study by the Groupe Francophone des Myélodysplasies (GFM). J. Clin. Oncol..

[156] Aprea Therapeutics (2020). Aprea Therapeutics Announces Results of Primary Endpoint from Phase 3 Trial of Eprenetapopt in TP53 Mutant Myelodysplastic Syndromes (MDS).

[157] Boeckler F.M., Joerger A.C., Jaggi G., Rutherford T.J., Veprintsev D.B., Fersht A.R. (2008). Targeted Rescue of a Destabilized Mutant of p53 by an in Silico Screened Drug. Proc. Natl. Acad. Sci. USA.

[158] Liu X., Wilcken R., Joerger A.C., Chuckowree I.S., Amin J., Spencer J., Fersht A.R. (2013). Small Molecule Induced Reactivation of Mutant p53 in Cancer Cells. Nucleic Acids Res..

[159] Hiraki M., Hwang S.-Y., Cao S., Ramadhar T.R., Byun S., Yoon K.W., Lee J.H., Chu K., Gurkar A.U., Kolev V. (2015). Small-Molecule Reactivation of Mutant p53 to Wild-Type-like p53 through the p53-Hsp40 Regulatory Axis. Chem. Biol..

[160] Nguyen D., Liao W., Zeng S.X., Lu H. (2017). Reviving the Guardian of the Genome: Small Molecule Activators of p53. Pharmacol. Ther..

[161] Schulz-Heddergott R., Stark N., Edmunds S.J., Li J., Conradi L.-C., Bohnenberger H., Ceteci F., Greten F.R., Dobbelstein M., Moll U.M. (2018). Therapeutic Ablation of Gain-of-Function Mutant p53 in Colorectal Cancer Inhibits Stat3-Mediated Tumor Growth and Invasion. Cancer Cell.

[162] Bossi G., Lapi E., Strano S., Rinaldo C., Blandino G., Sacchi A. (2006). Mutant p53 Gain of Function: Reduction of Tumor Malignancy of Human Cancer Cell Lines through Abrogation of Mutant p53 Expression. Oncogene.

[163] Yan W., Liu G., Scoumanne A., Chen X. (2008). Suppression of Inhibitor of Differentiation 2, a Target of Mutant p53, Is Required for Gain-of-Function Mutations. Cancer Res..

[164] Alexandrova E.M., Yallowitz A.R., Li D., Xu S., Schulz R., Proia D.A., Lozano G., Dobbelstein M., Moll U.M. (2015). Improving Survival by Exploiting Tumour Dependence on Stabilized Mutant p53 for Treatment. Nature.

[165] Li D., Marchenko N.D., Moll U.M. (2011). SAHA Shows Preferential Cytotoxicity in Mutant p53 Cancer Cells by Destabilizing Mutant p53 through Inhibition of the HDAC6-Hsp90 Chaperone Axis. Cell Death Differ..

[166] Pillai R.N., Fennell D.A., Kovcin V., Ciuleanu T.-E., Ramlau R., Kowalski D., Schenker M., Yalcin I., Teofilovici F., Vukovic V.M. (2019). Randomized Phase III Study of Ganetespib, a Heat Shock Protein 90 Inhibitor, with Docetaxel Versus Docetaxel in Advanced Non–Small-Cell Lung Cancer (GALAXY-2). J. Clin. Oncol..

[167] Blumenschein G.R., Kies M.S., Papadimitrakopoulou V.A., Lu C., Kumar A.J., Ricker J.L., Chiao J.H., Chen C., Frankel S.R. (2008). Phase II Trial of the Histone Deacetylase Inhibitor Vorinostat (Zolinza^TM^, Suberoylanilide Hydroxamic Acid, SAHA) in Patients with Recurrent and/or Metastatic Head and Neck Cancer. Invest. New Drugs.

[168] Martinez L.A., Naguibneva I., Lehrmann H., Vervisch A., Tchénio T., Lozano G., Harel-Bellan A. (2002). Synthetic Small Inhibiting RNAs: Efficient Tools to Inactivate Oncogenic Mutations and Restore p53 Pathways. Proc. Natl. Acad. Sci. USA.

[169] Ubby I., Krueger C., Rosato R., Qian W., Chang J., Sabapathy K. (2019). Cancer Therapeutic Targeting Using Mutant–p53-Specific SiRNAs. Oncogene.

[170] Debbas M., White E. (1993). Wild-Type p53 Mediates Apoptosis by E1A, Which Is Inhibited by E1B. Genes Dev..

[171] Bischoff J.R., Kirn D.H., Williams A., Heise C., Horn S., Muna M., Ng L., Nye J.A., Sampson-Johannes A., Fattaey A. (1996). An Adenovirus Mutant That Replicates Selectively in p53-Deficient Human Tumor Cells. Science.

[172] Heise C., Sampson-Johannes A., Williams A., Mccormick F., Von Hoff D.D., Kirn D.H. (1997). ONYX-015, an E1B Gene-Attenuated Adenovirus, Causes Tumor-Specific Cytolysis and Antitumoral Efficacy That Can Be Augmented by Standard Chemotherapeutic Agents. Nat. Med..

[173] Goodrum F.D., Ornelles D.A. (1998). p53 Status Does Not Determine Outcome of E1B 55-Kilodalton Mutant Adenovirus Lytic Infection. J. Virol..

[174] Rothmann T., Hengstermann A., Whitaker N.J., Scheffner M., zur Hausen H. (1998). Replication of ONYX-015, a Potential Anticancer Adenovirus, Is Independent of p53 Status in Tumor Cells. J. Virol..

[175] Rogulski K.R., Freytag S.O., Zhang K., Gilbert J.D., Paielli D.L., Kim J.H., Heise C.C., Kirn D.H. (2000). In Vivo Antitumor Activity of ONYX-015 Is Influenced by p53 Status and Is Augmented by Radiotherapy. Cancer Res..

[176] Nemunaitis J., Khuri F., Ganly I., Arseneau J., Posner M., Vokes E., Kuhn J., McCarty T., Landers S., Blackburn A. (2001). Phase II Trial of Intratumoral Administration of ONYX-015, a Replication-Selective Adenovirus, in Patients with Refractory Head and Neck Cancer. J. Clin. Oncol..

[177] Kirn D. (2001). Clinical Research Results with Dl1520 (Onyx-015), a Replication-Selective Adenovirus for the Treatment of Cancer: What Have We Learned?. Gene Ther..

[178] Reid T., Galanis E., Abbruzzese J., Sze D., Wein L.M., Andrews J., Randlev B., Heise C., Uprichard M., Hatfield M. (2002). Hepatic Arterial Infusion of a Replication-Selective Oncolytic Adenovirus (Dl1520). Cancer Res..

[179] Khuri F.R., Nemunaitis J., Ganly I., Arseneau J., Tannock I.F., Romel L., Gore M., Ironside J., MacDougall R.H., Heise C. (2000). A Controlled Trial of Intratumoral ONYX-015, a Selectively-Replicating Adenovirus, in Combination with Cisplatin and 5-Fluorouracil in Patients with Recurrent Head and Neck Cancer. Nat. Med..

[180] Garber K. (2006). China Approves World’s First Oncolytic Virus Therapy for Cancer Treatment. JNCI J. Natl. Cancer Inst..

[181] Weissmueller S., Manchado E., Saborowski M., Morris J.P., Wagenblast E., Davis C.A., Moon S.-H., Pfister N.T., Tschaharganeh D.F., Kitzing T. (2014). Mutant p53 Drives Pancreatic Cancer Metastasis through Cell-Autonomous PDGF Receptor β Signaling. Cell.

[182] Di Agostino S., Strano S., Emiliozzi V., Zerbini V., Mottolese M., Sacchi A., Blandino G., Piaggio G. (2006). Gain of Function of Mutant p53: The Mutant p53/NF-Y Protein Complex Reveals an Aberrant Transcriptional Mechanism of Cell Cycle Regulation. Cancer Cell.

[183] Welti J., Sharp A., Brooks N., Yuan W., McNair C., Chand S.N., Pal A., Figueiredo I., Riisnaes R., Gurel B. (2021). Targeting the P300/CBP Axis in Lethal Prostate Cancer. Cancer Discov..

[184] Lasko L.M., Jakob C.G., Edalji R.P., Qiu W., Montgomery D., Digiammarino E.L., Hansen T.M., Risi R.M., Frey R., Manaves V. (2017). Discovery of a Selective Catalytic P300/CBP Inhibitor That Targets Lineage-Specific Tumours. Nature.

[185] Yang Y., Zhang R., Li Z., Mei L., Wan S., Ding H., Chen Z., Xing J., Feng H., Han J. (2020). Discovery of Highly Potent, Selective, and Orally Efficacious P300/CBP Histone Acetyltransferases Inhibitors. J. Med. Chem..

[186] Capaci V., Mantovani F., Del Sal G. (2021). Amplifying Tumor–Stroma Communication: An Emerging Oncogenic Function of Mutant p53. Front. Oncol..

[187] Dong Z.-Y., Zhong W.-Z., Zhang X.-C., Su J., Xie Z., Liu S.-Y., Tu H.-Y., Chen H.-J., Sun Y.-L., Zhou Q. (2017). Potential Predictive Value of TP53 and KRAS Mutation Status for Response to PD-1 Blockade Immunotherapy in Lung Adenocarcinoma. Clin. Cancer Res..

[188] Sun H., Liu S.-Y., Zhou J.-Y., Xu J.-T., Zhang H.-K., Yan H.-H., Huan J.-J., Dai P.-P., Xu C.-R., Su J. (2020). Specific TP53 Subtype as Biomarker for Immune Checkpoint Inhibitors in Lung Adenocarcinoma. eBioMedicine.

[189] Asgari A., Lesyk G., Poitras E., Govindasamy N., Terry K., To R., Back V., Rudzinski J.K., Lewis J.D., Jurasz P. (2021). Platelets Stimulate Programmed Death-Ligand 1 Expression by Cancer Cells: Inhibition by Anti-Platelet Drugs. J. Thromb. Haemost..

[190] Larkin J., Chiarion-Sileni V., Gonzalez R., Grob J.-J., Rutkowski P., Lao C.D., Cowey C.L., Schadendorf D., Wagstaff J., Dummer R. (2019). Five-Year Survival with Combined Nivolumab and Ipilimumab in Advanced Melanoma. N. Engl. J. Med..

[191] Brown D.W., Raturi A., Bhandari P., Sosnowski D., Grin L., Wee P., Vega H., Gyoba J., Hejazi M., Ablack J. (2020). Selective Ablation of Solid Tumors Using a p53-Targeted FAST-LNP Gene Therapy. Cancer Res..

[192] Urist M., Tanaka T., Poyurovsky M.V., Prives C. (2004). P73 Induction after DNA Damage Is Regulated by Checkpoint Kinases Chk1 and Chk2. Genes Dev..

[193] Gottifredi V., Karni-Schmidt O., Shieh S.S., Prives C. (2001). p53 Down-Regulates CHK1 through P21 and the Retinoblastoma Protein. Mol. Cell Biol..

[194] Sachdeva M., Zhu S., Wu F., Wu H., Walia V., Kumar S., Elble R., Watabe K., Mo Y.-Y. (2009). p53 Represses C-Myc through Induction of the Tumor Suppressor MiR-145. Proc. Natl. Acad. Sci. USA.

[195] Shao J., Fujiwara T., Kadowaki Y., Fukazawa T., Waku T., Itoshima T., Yamatsuji T., Nishizaki M., Roth J.A., Tanaka N. (2000). Overexpression of the Wild-Type p53 Gene Inhibits NF-ΚB Activity and Synergizes with Aspirin to Induce Apoptosis in Human Colon Cancer Cells. Oncogene.

[196] Bos J.L. (1989). Review Ras Oncogenes in Human Cancer: A Review. Cancer Res..

[197] Arpaia E., Blaser H., Quintela-Fandino M., Duncan G., Leong H.S., Ablack A., Nambiar S.C., Lind E.F., Silvester J., Fleming C.K. (2012). The Interaction between Caveolin-1 and Rho-GTPases Promotes Metastasis by Controlling the Expression of Alpha5-Integrin and the Activation of Src, Ras and Erk. Oncogene.

[198] Grzes M., Oron M., Staszczak Z., Jaiswar A., Nowak-Niezgoda M., Walerych D. (2020). A Driver Never Works Alone—Interplay Networks of Mutant p53, MYC, RAS, and Other Universal Oncogenic Drivers in Human Cancer. Cancers.

[199] Ho J.S.L., Ma W., Mao D.Y.L., Benchimol S. (2005). p53-Dependent Transcriptional Repression of c-Myc Is Required for G 1 Cell Cycle Arrest. Mol. Cell Biol..

[200] Santoro A., Vlachou T., Luzi L., Melloni G., Mazzarella L., D’Elia E., Aobuli X., Pasi C.E., Reavie L., Bonetti P. (2019). p53 Loss in Breast Cancer Leads to Myc Activation, Increased Cell Plasticity, and Expression of a Mitotic Signature with Prognostic Value. Cell Rep..

[201] Buganim Y., Solomon H., Rais Y., Kistner D., Nachmany I., Brait M., Madar S., Goldstein I., Kalo E., Adam N. (2010). p53 Regulates the Ras Circuit to Inhibit the Expression of a Cancer-Related Gene Signature by Various Molecular Pathways. Cancer Res..

[202] Lu X. (2010). Tied up in Loops: Positive and Negative Autoregulation of p53. Cold Spring Harb. Perspect. Biol..

[203] Zeimet A.G., Marth C. (2003). Why Did p53 Gene Therapy Fail in Ovarian Cancer?. Lancet Oncol..

[204] Bessis N., GarciaCozar F.J., Boissier M.-C. (2004). Immune Responses to Gene Therapy Vectors: Influence on Vector Function and Effector Mechanisms. Gene Ther..

[205] Halbert C.L., Rutledge E.A., Allen J.M., Russell D.W., Miller A.D. (2000). Repeat Transduction in the Mouse Lung by Using Adeno-Associated Virus Vectors with Different Serotypes. J. Virol..

[206] Boutin S., Monteilhet V., Veron P., Leborgne C., Benveniste O., Montus M.F., Masurier C. (2010). Prevalence of Serum IgG and Neutralizing Factors against Adeno-Associated Virus (AAV) Types 1, 2, 5, 6, 8, and 9 in the Healthy Population: Implications for Gene Therapy Using AAV Vectors. Hum. Gene Ther..

[207] Nayak S., Herzog R.W. (2010). Progress and Prospects: Immune Responses to Viral Vectors. Gene Ther..

[208] Ferreira V., Twisk J., Kwikkers K., Aronica E., Brisson D., Methot J., Petry H., Gaudet D. (2014). Immune Responses to Intramuscular Administration of Alipogene Tiparvovec (AAV1-LPL(S447X)) in a Phase II Clinical Trial of Lipoprotein Lipase Deficiency Gene Therapy. Hum. Gene Ther..

[209] Masat E., Pavani G., Mingozzi F. (2013). Humoral Immunity to AAV Vectors in Gene Therapy: Challenges and Potential Solutions. Discov. Med..

[210] Mingozzi F., Maus M.V., Hui D.J., Sabatino D.E., Murphy S.L., Rasko J.E.J., Ragni M.V., Manno C.S., Sommer J., Jiang H. (2007). CD8+ T-Cell Responses to Adeno-Associated Virus Capsid in Humans. Nat. Med..

[211] Yin H., Kanasty R.L., Eltoukhy A.A., Vegas A.J., Dorkin J.R., Anderson D.G. (2014). Non-Viral Vectors for Gene-Based Therapy. Nat. Rev. Genet..

[212] Akinc A., Maier M.A., Manoharan M., Fitzgerald K., Jayaraman M., Barros S., Ansell S., Du X., Hope M.J., Madden T.D. (2019). The Onpattro Story and the Clinical Translation of Nanomedicines Containing Nucleic Acid-Based Drugs. Nat. Nanotechnol..

[213] Wan C., Allen T.M., Cullis P.R. (2014). Lipid Nanoparticle Delivery Systems for SiRNA-Based Therapeutics. Drug Deliv. Transl. Res..

[214] Polack F.P., Thomas S.J., Kitchin N., Absalon J., Gurtman A., Lockhart S., Perez J.L., Pérez Marc G., Moreira E.D., Zerbini C. (2020). Safety and Efficacy of the BNT162b2 MRNA Covid-19 Vaccine. N. Engl. J. Med..

[215] Baden L.R., El Sahly H.M., Essink B., Kotloff K., Frey S., Novak R., Diemert D., Spector S.A., Rouphael N., Creech C.B. (2021). Efficacy and Safety of the MRNA-1273 SARS-CoV-2 Vaccine. N. Engl. J. Med..

[216] Dumont A., Lohard S., Maillet L., Juin P.P., Barillé-Nion S. (2020). NOXA the BCL-2 Family Member behind the Scenes in Cancer Treatment. J. Cell. Signal..

[217] Montaño-Samaniego M., Bravo-Estupiñan D.M., Méndez-Guerrero O., Alarcón-Hernández E., Ibáñez-Hernández M. (2020). Strategies for Targeting Gene Therapy in Cancer Cells with Tumor-Specific Promoters. Front. Oncol..

[218] Liu J., Fu M., Wang M., Wan D., Wei Y., Wei X. (2022). Cancer Vaccines as Promising Immuno-Therapeutics: Platforms and Current Progress. J. Hematol. Oncol..

[219] Otto T., Sicinski P. (2017). Cell Cycle Proteins as Promising Targets in Cancer Therapy. Nat. Rev. Cancer.

[220] Ayoub N.M. (2021). Editorial: Novel Combination Therapies for the Treatment of Solid Cancers. Front. Oncol..

[221] Malone E.R., Oliva M., Sabatini P.J.B., Stockley T.L., Siu L.L. (2020). Molecular Profiling for Precision Cancer Therapies. Genome Med..

[222] Döhner H., Wei A.H., Löwenberg B. (2021). Towards Precision Medicine for AML. Nat. Rev. Clin. Oncol..

[223] Di Nicolantonio F., Vitiello P.P., Marsoni S., Siena S., Tabernero J., Trusolino L., Bernards R., Bardelli A. (2021). Precision Oncology in Metastatic Colorectal Cancer—From Biology to Medicine. Nat. Rev. Clin. Oncol..

